# Spatiotemporal Controllable Sono‐Nanovaccines Driven by Free‐Field Based Whole‐Body Ultrasound for Personalized Cancer Therapy

**DOI:** 10.1002/advs.202307920

**Published:** 2024-02-02

**Authors:** Yang Wang, Guangzhe Li, Jianlong Su, Yiming Liu, Xiaomai Zhang, Guanyi Zhang, Zhihao Wu, Jinrong Li, Yuxuan Zhang, Xu Wang, Zejia Yang, Ruimin Wang, Chengdong Wang, Liu Wang, Fangfang Sun, Weijie Zhao, Xuejian Wang, Xiaojun Peng, Kun Shao

**Affiliations:** ^1^ State Key Laboratory of Fine Chemicals School of Chemical Engineering Dalian University of Technology Dalian 116024 China; ^2^ State Key Laboratory of Fine Chemicals Department of Pharmacy School of Chemical Engineering Dalian University of Technology Dalian 116024 China; ^3^ Nuclear Medicine First Affiliated Hospital of Dalian Medical University Dalian 116021 China; ^4^ Department of Urology First Affiliated Hospital of Dalian Medical University Dalian 116021 China

**Keywords:** nanovaccines, tumor associated macrophages, tumor microenvironment modulation, ultrasound‐driven, whole‐body sonodynamic therapy

## Abstract

Therapeutic cancer vaccines fail to produce satisfactory outcomes against solid tumors since vaccine‐induced anti‐tumor immunity is significantly hampered by immunosuppression. Generating an in situ cancer vaccine targeting immunological cold tumor microenvironment (TME) appears attractive. Here, a type of free‐field based whole‐body ultrasound (US)‐driven nanovaccines are constructed, named G5‐CHC‐R, by conjugating the sonosensitizer, Chenghai *chlorin* (CHC) and the immunomodulator, resiquimod (R848) on top of a super small‐sized dendrimeric nanoscaffold. Once entering tumors, R848 can be cleaved from a hypoxia‐sensitive linker, thus modifying the TME via converting macrophage phenotypes. The animals bearing orthotopic pancreatic cancer with intestinal metastasis and breast cancer with lung metastasis are treated with G5‐CHC‐R under a free‐field based whole‐body US system. Benefit from the deep penetration capacity and highly spatiotemporal selectiveness, G5‐CHC‐R triggered by US represented a superior alternative for noninvasive irradiation of deep‐seated tumors and magnification of local immune responses via driving mass release of tumor antigens and “cold‐warm‐hot” three‐state transformation of TME. In addition to irradiating primary tumors, a robust adaptive anti‐tumor immunity is potentiated, leading to successful induction of systemic tumor suppression. The sono‐nanovaccines with good biocompatibility posed wide applicability to a broad spectrum of tumors, revealing immeasurable potential for translational research in oncology.

## Graphical abstract

The sono‐nanovaccines driven by whole‐body ultrasound irradiation may provide an antigen pool containing richer antigens, amplifying the immune responses via “cold‐warm‐hot” three‐state transformation of TME. Under a free‐field based whole‐body ultrasound system, the therapeutic efficacy of G5‐CHC‐R as well as their antitumor mechanisms are assessed in the animal models of orthotopic pancreatic cancer with intestinal metastasis and breast cancer with lung metastasis.

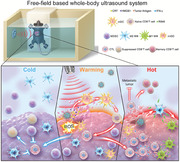

## 1 Introduction

Despite the paramount success, cancer immunotherapy maintains limited clinical benefits for patients with solid tumors owing to their immunosuppressive microenvironment.^[^ ^1^ ^]^ Solid tumors with poor immunogenicity are characterized by the lack of the host's pre‐existing anti‐tumor immunity and T lymphocytes infiltration, namely immunological “cold” tumors, which readily escape from immune clearance as well as induce progression and metastasis by modifying their local microenvironment.^[^ ^2^ ^]^ In the meantime, an abundance of various immunosuppressors such as tumor‐associated macrophages (TAMs), myeloid‐derived suppressor cells (MDSCs), and other suppressive factors in the non‐inflamed tumor microenvironment (TME), multifactorial immunosuppressive mechanisms inevitably occur, which hinder not only the natural host immune responses but also the efficacy of the immunotherapeutic interventions.^[^ ^3^ ^]^

Extensive efforts have been focused on therapeutic cancer vaccines, which strive to enhance tumor immunogenicity and cytotoxic T‐cell activity through precise delivery of large amounts of high‐quality tumor antigens and/or adjuvants.^[^ ^4^ ^]^ Despite vaccine technology being advanced at a breathtaking pace, the majority of therapeutic cancer vaccines still fail to realize their full potential.^[^ ^5^ ^]^ The lack of identified tumor antigen epitopes, low antigen delivery efficiency, as well as immune tolerogenic TME, remain major obstacles hindering the effectiveness and efficacy. Generating a vaccine in tumor situ by taking advantage of the whole repertoire of tumor antigens available at the tumor site has been proposed as a promising strategy.^[^ ^6^ ^]^ Emerging evidence has indicated that induction of immunogenic cell death (ICD) in tumor areas following certain treatments such as chemotherapy or radiotherapy allows for an ideal in situ vaccinal effect via facilitating the release of tumor‐associated antigens (TAAs), as well as triggering antigen‐specific cytotoxic T cell immune responses.^[^ ^7^ ^]^ Though TME could be warmed up via the induction of ICD, in most cases the cytotoxic approaches as monotherapy failed to fully prime the anti‐tumor immunity since the tumoricidal effector immune cells induced in situ were prone to turn dysfunctional in a relative cold TME, which posed negative impacts by providing the tolerogenic signals.^[^ ^8^ ^]^ For instance, large numbers of TAMs present in the TME suppress the activities of antigen‐presenting cells (APCs) and T cells via the release of an immunosuppressive cytokine, interleukin (IL)−10. In addition, MDSCs produce high levels of nitric oxide synthase and arginase, which inhibit the proliferation and activation of CD8^+^ T cells. Therefore, in situ vaccines primarily relying on immune responses initiation are inadequate, necessitating an appreciable microenvironment allowing for sufficient lymphocytic infiltration to induce enhanced and durable tumor remissions. Using nanoplatforms to integrate ICD induction as a trigger and immunosuppressive TME modulation to structure an in situ nano vaccine might offer a path to prime a broad anti‐tumor immunity most effectively.

Among all ICD‐inducible therapeutic modalities, sonodynamic therapy (SDT) represents a superior alternative. It employs low‐intensity ultrasound (US) to activate sonosensitizers to generate reactive oxygen species (ROS) to induce tumor cell apoptosis or necrosis, favoring various tumor‐associated antigens being exposed locally.^[^ ^9^ ^]^ Due to the minimal invasiveness, high spatiotemporally selectiveness, and deep penetration capacity, SDT has more advantages in the treatment of deep‐seated tumors.^[^ ^10^ ^]^ To synergize with SDT‐triggered therapeutic vaccination, modulating TAMs could be an effective strategy to rebuild the immunosuppressive TME.^[^ ^9^, ^11^ ^]^ Macrophages being the most abundant immune cells within tumors, are classified as antitumorigenic M1‐like or protumorigenic M2‐like macrophages.^[^ ^12^ ^]^ TAMs are thought to closely resemble M2‐like phenotype with major roles in tumor initiation, growth, development, and metastasis.^[^ ^13^ ^]^

As a potent TLR7/8 agonist, resiquimod (R848) has been approved by the FDA as a topical formulation for the treatment of cutaneous T cell lymphoma (CTCL) (NCT01676831) and basal cell carcinoma (NCT01808950) as monotherpy, or as vaccine adjuvants in patients with a variety of solid tumors such as melanoma (NCT00470379, NCT00821652, NCT02126579) and brain tumors (NCT01204684) in clinical trials.^[^ ^14^ ^]^ Many preclinical studies showed encouraging anti‐tumor activity via direct intratumoral (IT) delivery of R848,^[^ ^15^ ^]^ via recruiting and activating APCs, as well as promoting the release of pro‐inflammatory cytokines and chemokines.^[^ ^15^, ^16^ ^]^ In addition, R848 can also repolarize macrophages from M2‐like to M1‐like phonotype and deplete of MDSCs,^[^ ^15^, ^17^ ^]^ thereby positively regulating the immune networks. However, systemic R848 exposure and transit should be highly avoided due to undesired systemic risks, such as high levels of peripheral proinflammatory cytokines, thus increasing the risk of cytokine‐driven adverse effects,^[^ ^18^ ^]^ further highlighting the importance of nanoparticulated R848 for potentially preventing its off‐target effect.^[^ ^19^ ^]^

**Figure 1:**
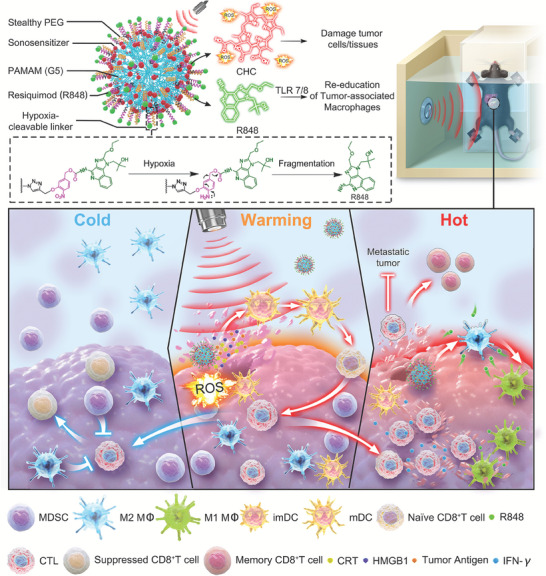
Schematic illustration of the structure of the sono‐nanovaccines and their therapeutical mechanisms driven by the free‐field based whole‐body US irradiation. Scheme of the sono‐nanovaccines, G5‐CHC‐R (upper), and schematic diagram of the sono‐nanovaccines for direct tumor cells killing as well as amplifying the cascade of the immune responses via “cold‐warm‐hot” three‐state transformation of TME (lower). MDSC, myeloid‐derived suppressor cell; M2 M*Φ*, M2‐phenotypic macrophage; M1 M*Φ*, M1‐phenotypic macrophage; imDC, immature dendritic cell; mDC, mature dendritic cell; CTL, cytotoxic T lymphocyte; CRT, calreticulin; HMGB1, high mobility group box protein 1.

To conduct US irradiation, we previously established a low‐intensity free‐field‐based ultrasonic system using water as a medium.^[^ ^9a^ ^]^ This free‐field ultrasonic system produces stable ultrasonic waves, covering the whole body of the animal being treated, termed whole‐body SDT. In comparison with focused US systems, many advantages have been offered, including good reproducibility and less mechanical damage to normal tissues. Herein in this article, we engineered one type of free‐field based whole‐body US‐driven in situ nanovaccines by using a super small‐sized dendrimeric nanoscaffold, the fifth generation of poly(amidoamine) (PAMAM, G5) as a vaccine platform. Previously in our laboratory, a porphyrinic derivative with an identical chemical structure to chlorin *e6* (Ce6) was successfully prepared from the natural spirulina powders originating in Chenghai Lake in Yunnan Province, China. This porphyrinic derivative, named Chenghai chlorin (CHC), showing high sono‐sensitive activity, excellent tumor targetability, and good biocompatibility was chemically modified on G5 surface.^[^ ^9^, ^20^ ^]^ Subsequently, the immunomodulator R848 was conjugated with G5 via a hypoxia‐sensitive linker, being cleavable by nitroreductase (NTR) highly expressed in tumor tissues.^[^ ^21^ ^]^ Due to the ultrasmall size, the nano vaccines G5‐CHC‐R exhibited excellent tumor target and penetration capacities. Under free‐field based whole‐body US irradiation, the sono‐nanovaccines G5‐CHC‐R generated ROS that induced ICD and TAAs to turn “cold” tumors into immune‐silent “warm” tumors. Hypoxic conditions cleaved the linker to unleash R848 to further enhance the anti‐tumor immunity by repolarizing the TAMs, down‐regulating MDSCs, and potentiating the cytotoxic responses of CD8^+^ T cells, ultimately modifying the tumors into hot. In both models of orthotopic pancreatic cancer with intestinal metastasis and breast cancer with lung metastasis, the sono‐nanovaccines significantly controlled primary tumor growth as well as distant metastasis via mounting potent anti‐tumor immune responses globally by enabling “cold‐warm‐hot” three‐state transformation in TME (**Figure** **1**).

## 2 Results

### 2.1 Synthesis and Characterization of G5‐CHC‐R

**Figure 2:**
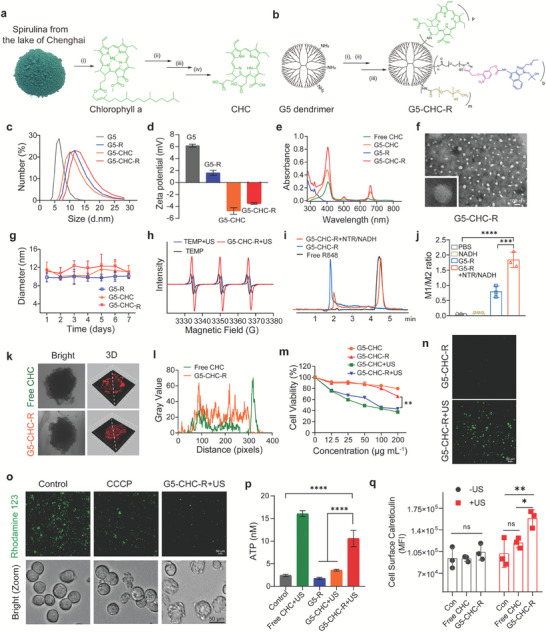
Schematic and characterization of the US‐driven sono‐nanovaccines, G5‐CHC‐R. a) Schematic illustration of the preparation of CHC. (i) Acetone, Reflux, 2 h. (ii) Et_2_O, HCl, yield 1.1%. (iii) 5% H_2_SO_4_ in CH_3_OH, 25 °C, 4 h; CH_3_OH, CH_3_ONa, 25 °C, 12 h, yield 23%. (iv) 5% KOH, THF, 40 °C, 12 h, yield 81%. b) Snythetic illustration of G5‐CHC‐R. (i) CHC, HOBt, EDC, 2 h. (ii) R848‐NTR‐COOH, NHS, EDC, 12 h. (iii) Methoxyl PEG carboxyl (Mw 2000), NHS, EDC, 12 h. Conjugation numbers, p = 14–18 per G5, b = 17–20 per G5. c,d) Hydrodynamic diameters in PBS pH 7.4 (c) and zeta potentials in water (d) of G5, G5‐R, G5‐CHC and G5‐CHC‐R measured by DLS. e) UV–vis absorbance spectra of free CHC, G5‐CHC, G5‐R and G5‐CHC‐R. f) TEM image showing the spherical morphology of G5‐CHC‐R with good dispersity. Scale bars, 100 nm. g) Long‐term stability of G5‐R, G5‐CHC, and G5‐CHC‐R in PBS pH 7.4 at room temperature for 7 days (n = 3). h) ESR spectra of G5‐CHC‐R with US irradiation. i) HPLC analysis demonstrating the release of R848 from G5‐CHC‐R triggered by NRT (100 µg mL^−1^) and NADH (100 µM). j) Ratios of M1 versus M2 phenotypic macrophages measured by flow cytometry (n = 3). k) CLSM images of 3D tumor spheroids after incubating with free CHC and G5‐CHC‐R at CHC concentration of 50 µg mL^−1^ for 4 h (Ex 559 nm, Em 650–700 nm, scale bar, 50 µm). l) Line‐scanning profiles showing the gray value indicated by the white lines shown in (k). m) Evaluation of sonotoxicity of G5‐CHC and G5‐CHC‐R against 4T1 cells by MTT assay. In vitro US irradiation condition was 1 MHz, 3 W cm^−2^ for 20 min (n = 6). n) Detecting ROS generation on 4T1 cells with an intracellular ROS‐specific probe, DCFH‐DA (Ex 450–490 nm, Em 500–550 nm, scale bar, 50 µm). o) The changes of mitochondrial membrane potentials of 4T1 cells after being treated with G5‐CHC‐R with US, stained with Rhodamine 123 (Ex 450–490 nm, Em 500–550 nm, scale bar, 50 µm). CCCP was set as positive control. p,q) 4T1 cells were incubated with free CHC, G5‐R, G5‐CHC, and G5‐CHC‐R for 4 h then following the US treatment (1 MHz, 3 W cm^−2^) for 20 min. ATP levels in the supernatants were analyzed as in (p) (n = 3) and CRT exposure was analyzed by flow cytometry in (q) (n = 3). Data represented mean ± SD. Statistical significance was calculated via Student's t‐test (m) or one‐way ANOVA with Dunnett's multiple comparison test (j,p,q); ns means no significant difference. *p*‐value: ^*^ *p* <0.05, ^**^ *p* <0.01, ^***^ *p* <0.001, and ^****^ *p* <0.0001.

The free‐field based whole‐body US‐driven sono‐nanovaccines were constructed with the fifth generation of PAMAM dendrimers (G5), which served as a highly branched nano‐sized scaffold for the co‐delivery of the sonosensitizer, CHC and the immune modulator, R848 (Figure 1). CHC was prepared from the carboxyl methyl esterified product of Chenghai pheophorbide a (CHP), which was converted from chlorophyll a crude extracted from Chenghai spirulina powders (**Figure** **2**a; Figures S1**–**S6, Supporting Information). The covalent conjugation of CHC onto the scaffold was via the condensation reaction between the amino groups on G5 and the carboxylate group at position 15 of CHC (Figure 2b; Figures S15 and S16, supporting Information). A hypoxia‐cleavable linker was integrated into the nanosystem to bridge R848 and G5, to achieve the tumor‐specific drug deposited (Figures S7–S14, Supporting Information). For comparison, the CHC‐ or R848‐ single conjugated nanoparticles (G5‐CHC or G5‐R) and the methoxy peglated PAMAM (G5) were prepared (Figures S16 and S17, Supporting Information). The average conjugation numbers were calculated as 14–18 CHC per G5 and/or 17–20 R848 per G5 (Figure 2b; Figures S15–S20, Supporting Information). The modifications with CHC and/or R848 increased the average hydrodynamic diameter of G5 from 9.82 to 12.26 nm approximately (Figure 2c). Both G5‐CHC and G5‐CHC‐R exhibited a highly negative zeta potential due to the free carboxyl groups of CHC, −4.763 ± 0.56 and −3.503 ± 0.13 mV, respectively (Figure 2d). The distinct absorption peaks at 325 nm (R848), 404 and 665 nm (CHC) were observed in the UV–vis absorption spectrum of G5‐CHC‐R (Figure 2e), indicating the successful conjugation of R848 and CHC. Remarkably, compared with free CHC, G5‐CHC, and G5‐CHC‐R had a broader and redshifted Soret band due to the improved water solubility of CHC after conjugation onto the nanoparticles (Figure 2e). Analysis using transmission electron microscopy (TEM) showed that G5‐CHC‐R nanoparticles were well dispersed with spherical morphology (Figure 2f). G5‐R, G5‐CHC, and G5‐CHC‐R retained a uniform size distribution without obvious variations in PBS for 7 days (Figure 2g), revealing excellent long‐term stability. The US‐triggered ROS generation capacities of CHC and G5‐CHC‐R in the solution were assayed with one commercial ROS‐sensing agent, 1,3‐diphenylisobenzofuran (DPBF) that could be bleached to the corresponding diketone once interacting with ROS. As US irradiation time increased, the relative intensities of DPBF (maximum absorption wavelength 407 nm) in both free CHC+US and G5‐CHC‐R+US treated groups showed significant decrease, suggesting the negligible impacts on sonosensitive activity of CHC after conjugating to G5 (Figure S21, Supporting Information). In the meantime, ROS species triggered by G5‐CHC‐R during US irradiation were monitored by electron spin resonance (ESR) assay as well. The remarkable characteristic peaks of ^1^O_2_ species were observed in G5‐CHC‐R+US treated group, further confirming the sono‐sensitive activity of G5‐CHC‐R (Figure 2h).

The dissociation of R848 from G5‐CHC‐R catalyzed by over‐expression of NTR was validated. High‐performance liquid chromatography (HPLC) analysis was conducted after incubating G5‐CHC‐R with NTR/nicotinamide adenine dinucleotide (NADH) at 37 °C for 4 h. An obvious peak at 4.873 min corresponding to free R848 was observed in the elution profile (Figure 2i), indicating the cleavability of the linker by NTR catalysis. The effect of released R848 on macrophage phenotypic conversion was further confirmed in vitro. Mouse bone marrow‐derived macrophages (BMDMs) were first polarized to an M2 phenotype upon 24 h of treatment with IL‐4. The group incubated with G5‐R+NTR/NADH showed a remarkable M2‐to‐M1 phenotype switching (Figure 2j; Figure S22, Supporting Information), similar to the observation in free R848 treated groups (Figure S23, Supporting Information). In contrast, the transformation efficacy was significantly shielded once being co‐incubated without NTR/NADH. This might be because R848 lost partial activity after chemical modification on C4 amino group,^[^ ^22^ ^]^ suggesting the necessity of NTR‐triggered release of R848 from the vehicles.

Efficient tumor infiltration and accumulation is the premise for a good therapeutic outcome. We therefore evaluated the cellular uptake of G5‐CHC‐R on 4T1 and Pan02 tumor cell lines, respectively. The red fluorescence signals inside the cells were gradually enhanced as incubation time increased (1, 4, 8, and 12 h) (Figures S24 and S25, Supporting Information), indicating the efficient internalization and extended cellular retention of G5‐CHC‐R. Images of 4T1 tumor spheroid taken by confocal laser scanning microscopy (CLSM) showed that free CHC was mainly lodged in the outer rims of the spheroid after 4 h incubation, whereas G5‐CHC‐R had been distributed uniformly throughout the spheroids (Figure 2k,l). Both CHC and G5‐CHC‐R showed negligible cytotoxicity on 3D tumor spheroids at the concentration of 50 µg mL^−1^ of CHC, making sure that the excellent tumor penetrability of G5‐CHC‐R was not caused by cell death in the tumor spheroids (Figure S26, Supporting Information). Benefiting from the optimal smaller size (<20 nm),^[^ ^23^ ^]^ G5‐CHC‐R enabled notably better tumor permeability, resulting in higher accumulation levels of the cargoes in vivo at tumor sites (Figure S27, Supporting Information).

MTT assay was conducted to verify the SDT effectiveness of G5‐CHC‐R in vitro on 4T1 cells. In the absence of US, both G5‐CHC and G5‐CHC‐R exhibited negligible cytotoxicity, suggesting their favorable biocompatibilities (Figure 2m). Notably, after being irradiated by the US (1 MHz, 3 W cm^−2^) for 20 min, the cell viabilities were significantly decreased to 37.33% and 43.03% (Figure 2m), revealing the excellent cell‐killing effect, which was well associated with a higher level of intracellular ROS production induced by G5‐CHC‐R under US irradiation (Figure 2n). As ROS production is eventually accompanied with mitochondrial damage and dysfunction,^[^ ^24^ ^]^ we next investigated the changes of mitochondrial membrane potentials by using Rhodamine 123. Treatment with CCCP (Carbonyl cyanide 3‐chlorophenylhydrazone) was set as positive control. The remarkable reduction of green signals was observed in the G5‐CHC‐R+US treated group, indicating the loss of mitochondrial membrane integrity (Figure 2o). Consistently being observed in the bright images, the obvious morphologic transformation suggested that the cells initiated mitochondrial apoptosis caused by the accumulated ROS (Figure 2o).^[^ ^25^ ^]^ In addition, excessive intracellular ROS can drive cancer cells to go through ICD as well, which is characterized by actively emitting damage‐associated molecular patterns (DAMPs) such as surface‐exposed calreticulin (CRT) and secreted ATP.^[^ ^26^ ^]^ The results of CRT expression and ATP secretion were further confirmed the ICD‐inducible capabilities of G5‐CHC‐R in the presence of US (Figure 2p,q; Figure S28, Supporting Information), which was crucial for subsequently activating the potent anticancer immunity. Taken together, all these data revealed the excellent acoustic responsive activity of G5‐CHC‐R, suggesting its great potential for SDT application in vivo.

### 2.2 Inhibition of Orthotopic Pancreatic Cancer Growth

**Figure 3:**
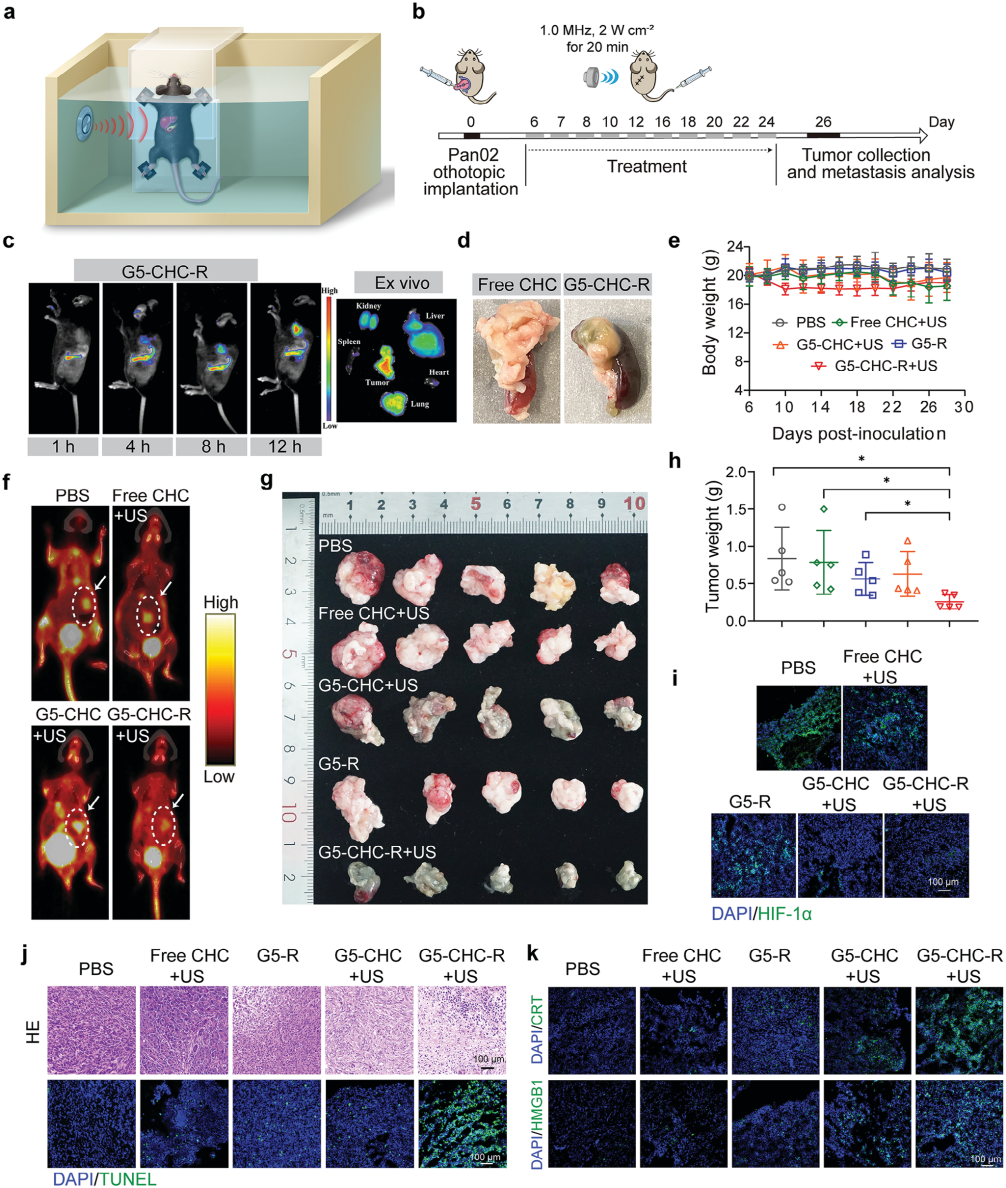
The sono‐nanovaccines driven by the free‐field‐based whole‐body US system inhibited primary tumor growth in the orthotopic pancreatic cancer model with intestinal metastasis. a) Schematic illustration of equipment setup for free‐field based whole‐body US irradiation in vivo. b) Time course for the experimental design. A murine pancreatic cancer model with intestinal metastasis was established via orthotopic inoculation of 5 × 10^5^ Pan02 cells per mouse. Different groups were intravenous administration with PBS, free CHC, G5‐CHC, G5‐R, and G5‐CHC‐R at 15 mg CHC kg^−1^ body weight (for G5‐R, 1 mg R848 kg^−1^ body weight). For the groups with US irradiation, the mice were kept in the whole‐body US system shown in (a) to receive irradiation at 1.0 MHz, 2 W cm^−2^ for 20 min. c) In vivo real‐time fluorescence imaging of model mice after intravenous injection of G5‐CHC‐R at different time points, as well as the ex vivo tissue images after 12 h post‐injection (Ex 665 nm, Em 700 nm). d) The photograph of the pancreatic tumor indicated color change after three consecutive doses of G5‐CHC‐R. e) The body weights of different groups with various treatments (n  =  5). f) Noninvasive evaluation of primary tumor burdens in the pancreas by micro PET/CT (coronal view). g) The photographs of the excised pancreatic tumors at the endpoint (n  =  5). h) Average weights of excised primary pancreatic tumors of different treated groups (n  =  5). i,k) Immunofluorescence staining with HIF‐1α (i), CRT, and HMGB1 (k) in the excised tumor tissues after various treatments. Scale bar, 100 µm. j) Representative H&E and TUNEL images of tumor sections. Scale bar, 100 µm. Data represented mean ± SD. Statistical significance was calculated via Student's t‐test (h) or one‐way ANOVA with Dunnett's multiple comparison test; *p*‐value: ^*^ *p* < 0.05, ^**^ *p* < 0.01, ^***^ *p* < 0.001, and ^****^ *p* <0.0001.

To assess the synergistic therapeutic effect of the free‐field based whole‐body US‐driven sono‐nanovaccines, a murine Pan02‐orthotopic pancreatic cancer model with colon metastasis was established.^[^ ^27^ ^]^ The majority of therapeutics failed to enter pancreatic tumor tissues due to a strong barrier formed by the desmoplastic stroma.^[^ ^28^ ^]^ Nevertheless, we found that G5‐CHC‐R preferentially localized to the pancreatic tumor site and retained at a predominant level until 12 h after I.V. injection (**Figure** **3**c). In addition, the dark green color derived from CHC was visually observed in the pancreatic tumors of mice treated with three consecutive doses of G5‐CHC‐R, but not with free CHC (Figure 3d), indicating the potential in overcoming the drug‐entry barriers as well as excellent tumor accumulation capacity of G5‐CHC‐R.

The tumor‐bearing mice were randomly divided into five groups: PBS, free CHC+US, G5‐R, G5‐CHC+US, and G5‐CHC‐R+US. The groups I.V. administrated with free CHC, G5‐CHC or G5‐CHC‐R were further exposed to the free‐field based whole‐body covered US irradiation (1.0 MHz, 2 W cm^−2^) for 20 min at 4 h post‐injection. The effectiveness and safety of the US irradiation condition were optimized in the mice bearing deep‐seated tumors in our previous study.^[^ ^9a^ ^]^ The US irradiation set‐up for the in vivo study and dosing regimen were presented in Figure 3a,b. Throughout the treatment, the body weights of the mice maintained stable (Figure 3e). To analyze hemolysis, the defibrotic sheep plasma treated with the highest concentration of G5‐CHC‐R containing up to 500 µg CHC mL^−1^ showed negligible hemolysis, with clear observation of the blood cell pallets in the bottom of the tube, whereas the group treated with distilled de‐ionized water (ddH_2_O) was fully hemolyzed (Figure S29, Supporting Information). In addition, the sono‐nanovaccines were able to be gradually cleared from the major organs through liver metabolism possibly (Figure S30, Supporting Information), resulting in less retention in non‐specific tissues. This could be the reason that there was no obvious destructive cell necrosis observed in the histological analysis of different major organs (Figure S31, Supporting Information). Collectively, all these results verified the safety and tolerability of free‐field‐based whole‐body US irradiation applied in our study, as well as the good biocompatibility of the sono‐nanovaccines in vivo.

On day 24 after tumor implantation, the tumor burden in the pancreas was evaluated by using positron emission tomography/computed tomography (PET/CT) for small animals. The lowest uptake of ^18^F‐fibroblast activation protein inhibitor (^18^F‐FAPI) was observed in the G5‐CHC‐R+US treated group (Figure 3f). Correlating well with the results of micro PET/CT, the analysis of average tumor weights confirmed that G5‐CHC‐R+US exhibited a profound attenuation in pancreatic tumor growth, whereas the groups of G5‐CHC+US and free CHC+US only showing a mild tumor inhibitory effect (Figure 3g,h), revealing the essentials of synergism. In addition, combinational therapy mediated by G5‐CHC‐R+US demonstrated significant alterations in tumor cell morphology, including nuclear pyknosis (Figure 3j, upper), fragmentation, and nucleolysis, as well as remarkable elevation of TUNEL‐positive cells in tumor sections (Figure 3j, lower), which together corresponded to extensive cell apoptosis and necrosis.

It is well established that intratumoral hypoxia increases hypoxia‐inducible factor 1 alpha (HIF‐1α) protein levels, which correlates with facilitated tumor growth and metastasis.^[^ ^29^ ^]^ ROS has been reported to downregulate the activity of HIF‐1α via inhibiting HIF‐1 DNA‐binding activity and accumulation under hypoxia.^[^ ^30^ ^]^ Immunofluorescence staining of HIF‐1α in tumor sections showed the significant decreased signals in groups treated with G5‐CHC+US and G5‐CHC‐R+US (Figure 3i), suggesting an association with enhanced ROS levels. Of note, free CHC+US treatment showed little impact on tumor growth inhibition, cell damage induction, and HIF‐1α down‐expression (Figure 3i; Figure S32, Supporting Information), which meant that CHC enabled SDT responses only when delivered by our nanoplatform, as free administration of CHC had limited access to the pancreatic tumor site.

SDT‐induced ICD plays a crucial role in the success of initiating an antitumor adaptive immune response by promoting the maturation of dendritic cells (DCs).^[^ ^31^ ^]^ To assess this phenomenon, primary pancreatic tumor samples were collected from the different treated groups and analyzed via immunofluorescent staining. As shown in Figure 3k, G5‐CHC‐R+US treated group demonstrated significantly up‐regulated expression of the CRT and high mobility group box 1 (HMGB1) in tumor tissues, suggesting higher ICD‐inducing capacity.

### 2.3 “Cold‐Warm‐Hot” Three‐State Transformation of TME

**Figure 4:**
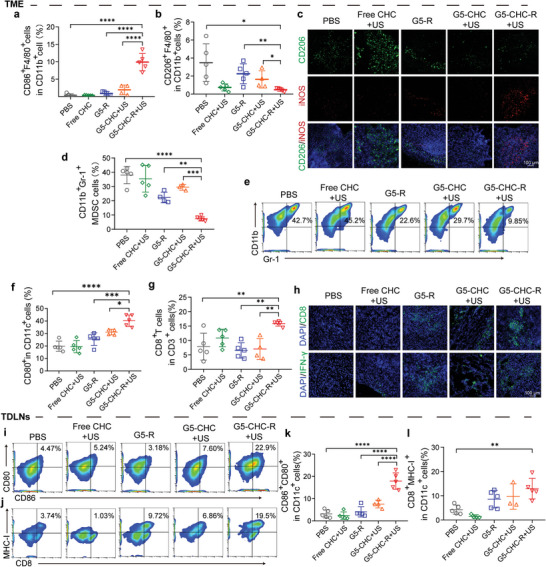
The free‐field‐based whole‐body US‐driven nanovaccines amplified the cascade of the immune responses in tumor situ via “cold‐warm‐hot” three‐state transformation of TME. a–c) Pancreatic tumor tissues were analyzed on day 26 for F4/80^+^CD86^+^ M1‐like (a) (n = 5) and F4/80^+^CD206^+^ M2‐like (b) (n = 5) macrophages measured by flow cytometry, and corresponding immunofluorescence images stained with indicated antibodies and DAPI (c). Scale bar, 100 µm. d,e) Number of CD11b^+^Gr‐1^+^ MDSC cells as a percentage of the total CD45^+^ leucocytes (d) (n = 5) and representative dot plots (e) in tumor tissues. f) Number of CD80^+^ DCs as a percentage of the total CD11c^+^ cell population in tumor tissues (n = 5). g) Number of CD8^+^ T cells as a percentage of the total CD3^+^ cell population in tumor tissues (n = 5). h) Immunofluorescence analysis of CD8^+^ T cells and IFN‐γ production on the tissue sections of the collected primary tumors. Scale bar, 100 µm. i–l) DCs in tumor‐draining lymph nodes were analyzed for their activated status (CD80^+^CD86^+^) and cross‐presenting capacities (MHC‐I^+^CD8^+^), shown as representative dot plots (i,j) and percentage analysis (k,l) (n = 5). Data represented mean ± SD. Statistical significance was calculated via Student's t‐test or one‐way ANOVA with Dunnett's multiple comparison test (a,b,d,f,g,k,l); *p*‐value: ^*^ *p* <0.05, ^**^ *p* <0.01, ^***^ *p* <0.001, and ^****^ *p* <0.0001.

To explore the underlying mechanisms by which the US‐driven sono‐nanovaccines inhibited primary pancreatic cancer growth, the immune landscape within TME was investigated. Pancreatic tumor exhibits an immunologically “cold” TME characterized by a prominent myeloid cell infiltration typically devoid of CD8^+^ effector T cells.^[^ ^32^ ^]^ The pro‐tumorigenic TAMs are one of the most abundant cellular components in pancreatic TME, heavily involved in immunosuppression. Thus, the phenotypic alteration of TAMs was first determined. As shown in **Figures** **4**a,b, and S33 (Supporting Information), TAMs were efficaciously polarized by G5‐CHC‐R+US treatment, evidenced by ≈27‐fold increase in M1‐phenotypic percentages and sevenfold decrease in M2‐phenotypic percentages (vs PBS treated group). This result was further verified by immunofluorescence using different markers to distinguish M1 (inducible nitric oxide synthase, iNOS) and M2 (mannose receptor, CD206) populations (Figure 4c). MDSCs are another pivotal suppressive factor in TME with a remarkable capacity to hinder immunological responses.^[^ ^15b^ ^]^ Similarly observed in our results, nanoparticulated R848 (G5‐R) could reduce MDSCs populations to some extent, nevertheless, the maximum depletion of MDSCs was achieved by the combinational therapy mediated by the sono‐nanovaccines (Figure 4d,e). Afterward, the localized antitumor immune responses were assessed. It was found that G5‐CHC‐R+US treatment induced highest levels of DCs maturation (40.3 ± 4.6%) and substantial expansion and infiltration of CD8^+^ T cells (15.5 ± 0.84%) (Figure 4f,g; Figures S34 and S35, Supporting Information), particularly tumoricidal IFN‐γ‐secreting CD8^+^ T cells (Figure. 4h). All these results demonstrated that the sono‐nanovaccines driven by US favorably altered the immune‐defective tumor (cold) into immunogenic and immunoreactive (hot) environment.

Tumor‐draining lymph nodes (TDLNs) are one of the crucial peripheral lymphoid organs where mature DCs cross‐present tumor antigens to the T lymphocytes to induce systemic anti‐tumor immunity.^[^ ^33^ ^]^ To examine the activated status and cross‐presentation of DCs, TDLNs were collected and analyzed via flow cytometry. The percentage of mature DCs (CD80^+^CD86^+^CD11c^+^) in the G5‐CHC‐R+US treated group (17.9 ± 3.7%) was significantly higher than all other groups (3.2 ± 1.8%, 2.4 ± 1.7%, 4.1 ± 2.3%, and 7.6 ± 1.7% for PBS, free CHC+US, G5‐R, and G5‐CHC+US, respectively) (Figure 4i,k; Figure S36, Supporting Information). In addition, G5‐CHC‐R+US treatment facilitated the expression of major histocompatibility complex class I (MHC‐I) molecules on CD8a^+^ DCs (Figure 4j,l), revealing the potentials in potentiation of peripheral CD8^+^ T cell responses.

### 2.4 Boosting Systemic Anti‐Tumor Immune Responses

**Figure 5:**
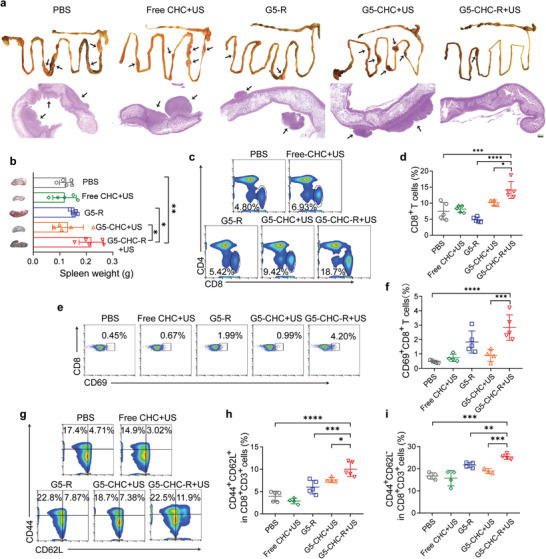
The free‐field‐based whole‐body US‐driven nano vaccines potentiated systemic anti‐tumor immune responses to attenuate intestinal metastasis. a) Representative photographs showing the gross appearance of tumor nodules in the intestines and corresponding H&E staining of tissue sections. Scale bar, 500 µm. b) Average weights of spleens for each group and corresponding representative photos (n = 5). c,d) Splenocytes from different treated groups were analyzed for percentages of CD8^+^ T cells in the total CD3^+^ cell population, shown as representative dot plots (c) and histograms (d) (n = 5). e,f) The representative dot plots (e) and histograms (f) show the expression of activation marker CD69 in CD8^+^ T cell population (n = 5). g–i) The representative dot plots (g) and histograms (h,i) show proportions of CD62L^+^CD44^+^ central memory T cells (T_CM_) and CD62L^−^CD44^+^ effector memory T cells (T_EM_) in CD8^+^ T cells in the spleens (n = 5). Data represented mean ± SD. Statistical significance was calculated via Student's t‐test or one‐way ANOVA with Dunnett's multiple comparison tests (b,d,f,h,i); *p*‐value: ^*^ *p* <0.05, ^**^ *p* <0.01, ^***^ *p* <0.001, and ^****^ *p* <0.0001.

Given the aggressively invasive feature of pancreatic cancer cells, the metastatic tumor burden was also a critical parameter for therapeutic evaluation. We could obviously see the metastatic seeding and outgrowth in the intestines of model mice (**Figure** **5**a). Consistent with the reliance on primary tumor inhibitory, G5‐CHC‐R+US treatment significantly reduced the tumor spread as well (Figure 5a). H&E staining showed that extensive areas of metastatic lesions were distributed in the mesenteries of all treated groups except G5‐CHC‐R+US (Figure 5a, lower). Remarkably, the slightly enlarged spleens and enhanced lymphocytic infiltration in tumor specimens were noted in G5‐CHC‐R+US treated group (Figure 5b). These observations implicated that the heightened host protective immune responses had been initiated by the US‐driven sono‐nanovaccines to oppose tumor development.

Motivated by the effective transformation of TME and the excellent anti‐metastatic effect induced by the US‐driven sono‐nanovaccines, we subsequently assessed its ability to boost systemic immune responses. In comparison to G5‐R and G5‐CHC+US treated groups, G5‐CHC‐R+US led to a 2.9‐ and 1.4‐fold increase in splenic CD8^+^ T cells (Figure 5c,d). Meanwhile, higher percentages of activated CD8^+^ T cells were observed in G5‐CHC‐R+US group, evidenced by the remarkable up‐regulated expression of activation marker CD69 (Figure 5e,f), suggesting the expansion and proliferation of cytotoxic T lymphocytes (CTLs) in the periphery.^[^ ^9a^ ^]^ Memory lymphocytes play an important role in mediating immune‐protection.^[^ ^34^ ^]^ Effector memory T cells (T_EM_, CD44^+^ CD62L^−^) execute immediate protection in peripheral tissues, whereas central memory T cells (T_CM_, CD44^+^ CD62L^+^) compasses the capabilities of mounting recall responses to antigenic stimulation.^[^ ^35^ ^]^ As shown in Figure 5g–i, G5‐CHC‐R+US treatment induced significant differentiation of peripheric CD8^+^ T cells into both effector and central memory phenotypes, suggesting a potential immunologic fortification established by the US‐driven sono‐nanovaccines, capable of preventing metastasis and conferring the long‐lasting immune protection against cancers.

### 2.5 Anti‐Tumor Activities and TME Alternations in a Spontaneous Breast Cancer with Lung Metastasis Model

**Figure 6:**
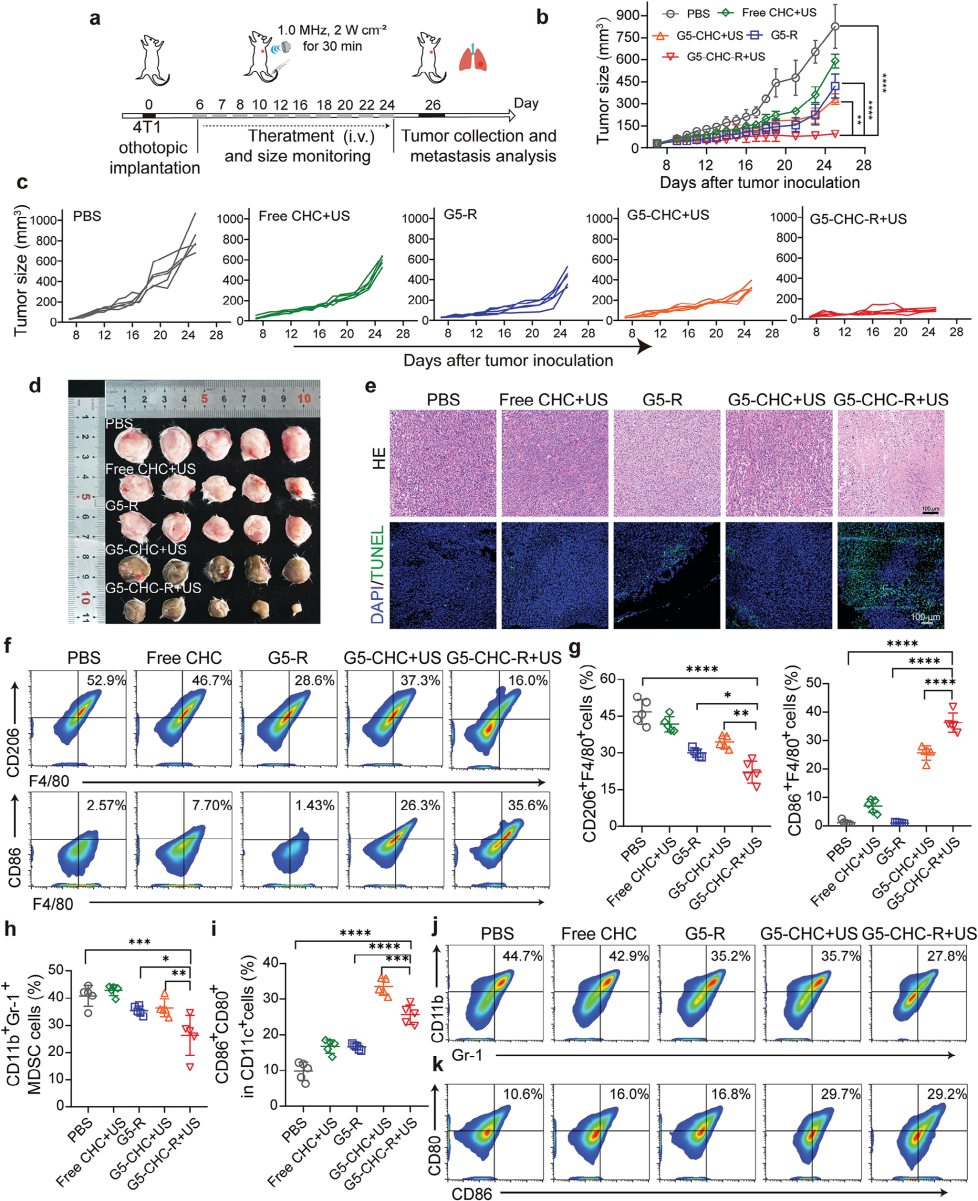
Anti‐tumor activities and TME alternations elicited by the free‐field based whole‐body US‐driven nano vaccines in spontaneous breast cancer with lung metastasis model. a) Time course for the experimental design. A murine breast cancer with spontaneous lung metastasis was established via injection of 5 × 10^5^ 4T1 cells into the second pair of mammary glands of female BALB/c mice. Different groups were intravenous administration with PBS, free CHC, G5‐CHC, G5‐R, and G5‐CHC‐R at 15 mg CHC kg^−1^ body weight (for G5‐R, 1 mg R848 kg^−1^ body weight). For the groups with US irradiation, the mice were kept in the free‐field based whole‐body US system to receive irradiation at 1.0 MHz, 2 W cm^−2^ for 30 min. b,c) Monitoring average tumor sizes (a) and **i**ndividual tumor growth curve (c) for each group versus days after tumor inoculation. d) Photographs of excised breast tumors at the endpoint. e) Representative images of tumor sections of different treated groups stained with H&E and TUNEL. Scale bar, 100 µm. f,g) Breast tumor tissues were analyzed on day 26 for F4/80^+^CD206^+^ M2‐like and F4/80^+^CD86^+^ M1‐like macrophages measured by flow cytometry, shown as representative dot plots (f) and histograms (g) (n = 5). h,j) Number of CD11b^+^Gr‐1^+^ MDSC cells as a percentage of the total CD45^+^ leucocytes (h) (n = 5) and representative dot plots (j) in tumor tissues. i,k) Number of CD86^+^CD80^+^ mature DCs as a percentage of the total CD11c^+^ cell population (i) (n = 5) and representative dot plots (k) in tumor tissues. Data represented mean ± SD. Statistical significance was calculated via Student's t‐test or one‐way ANOVA with Dunnett's multiple comparison tests (b, f‐k); *p*‐value: ^*^ *p* <0.05, ^**^ *p* <0.01, ^***^ *p* <0.001, and ^****^ *p* <0.0001.

To explore the general applicability of the US‐driven sono‐nano vaccines as a multimodal combinational therapeutic platform, we evaluated their effectiveness in breast cancer with lung metastasis model. In this model, murine breast cancer cells (4T1) being engrafted into the mammary glands of mice, spontaneously metastasized to the lungs.^[^ ^36^ ^]^ A total ten times of injections with different nanoparticles with or without US irradiation were conducted as shown in **Figure** **6**a. After three doses, the appearance of primary tumors of G5‐CHC‐R+US treated group was in dark green, but not free CHC+US group (Figure S37, Supporting Information), confirming the preferential tumor accumulation efficiency of the sono‐nanovaccines. The curves of tumor volumes showed that opposite to the uncontrollable growth in the PBS‐treated group, the treatment with free CHC+US, G5‐R, or G5‐CHC+US moderately delayed the tumor growth to some extent (Figure 6b,c). In stark contrast, G5‐CHC‐R+US treatment notably induced tumor regression with the lowest tumor weights (Figure 6b–d; Figure S38, Supporting Information). H&E and TUNEL staining were performed to detect tumor apoptosis in the primary tumor sites. Highly remarkable chromatin condensation, nuclear shrinkage, and expanded intercellular spaces were presented in the tumor sections of G5‐CHC‐R+US treated group (Figure 6e, upper). Consistently, G5‐CHC‐R+US treatment induced higher numbers of TUNEL‐positive apoptotic cells in tumor regions (Figure 6e, lower). All these findings further confirmed the anti‐tumor efficacy was greatly potentiated by the sono‐immuno combined modality.

Next, we investigated the impacts of the US‐driven sono‐nanovaccines on the composition of immune cells in the “cold” TME of breast cancer.^[^ ^37^ ^]^ As expected, the proportion of M2‐phenotypic macrophages in TME significantly reduced by 2.1‐ and 1.3‐fold when treated with G5‐CHC‐R+US, compared to the PBS and G5‐R treated groups (Figure 6f). In addition, the animals treated with G5‐CHC‐R+US presented the highest level of M1‐phenotypic macrophages as well as the notable reduction in the populations of immunosuppressive MDSCs (Figure 6f–h,j). Notably, a relative higher induction of M1‐phenotypic macrophages was found in G5‐CHC+US treated group, compared with G5‐R treated one (Figure 6f,g). Those results could possibly be ascribed to the positive impact on TME reversal posed by G5‐CHC‐mediated SDT, through increasing tumor immunogenicity to warm up the “cold” TME. And this effect was cascaded once in combination with R848. Benefited from reprogrammed TME, G5‐CHC‐R+US treatment prominently facilitated DCs maturation, 2.6‐fold higher than that of control group (Figure 6i,k), eventually expecting stronger anti‐tumor immune responses.

During the treatment course, neither weight losses nor significant organ damage were observed in any treated mice, indicating the biocompatibility of the different nanoparticles as well as the therapeutic modality (Figures S39 and S40, Supporting Information). Serum biochemistry indexes of alanine aminotransferase (ALT), aspartate aminotransferase (AST), and creatinine/carbamide urea (CRE/UREA) were assayed, assuring no liver and renal damages caused by the US‐driven sono‐nanovaccines (Figure S41, Supporting Information).

### 2.6 Synergistically Controlling Lung Metastasis via Systemic Anti‐Tumor Immunity

**Figure 7:**
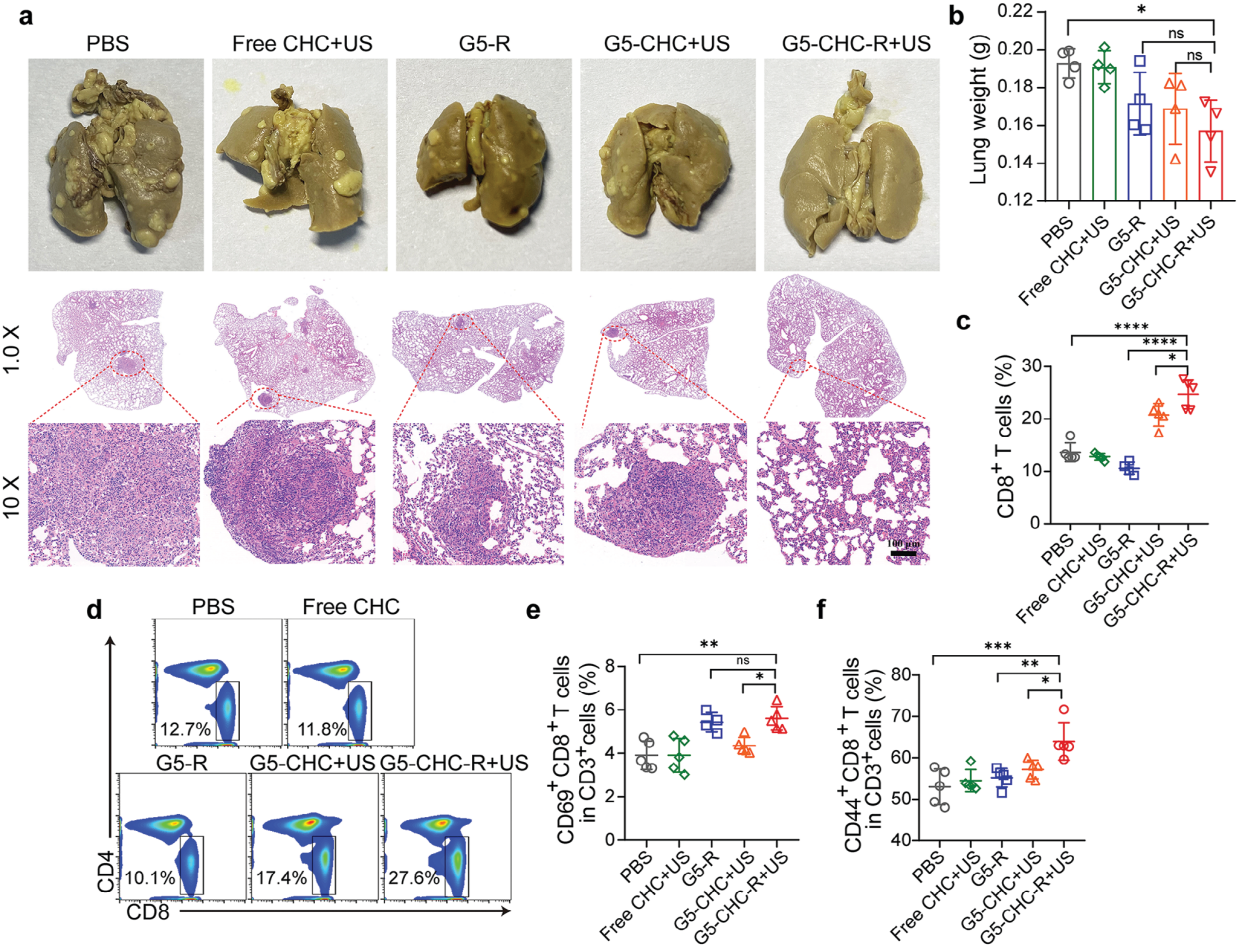
The free‐field based whole‐body US‐driven nanovaccines synergistically controlled lung metastasis via systemic anti‐tumor immunity. a) Representative photographs of excised lungs of different treated groups and corresponding tissue sections stained with H&E. b) Average lung weights measured at endpoint (n = 4). c,d) Splenocytes from different treated groups were analyzed for percentages of CD8^+^ T cells in the total CD3^+^ cell population, shown as histograms (c) (n = 5) and representative dot plots (d). e) The histograms showing the expression of activation marker CD69 in CD8^+^ T cell population in the spleens (n = 4). f) The histograms showing the persentages of CD44^+^ memory T cells in CD8^+^ T cell population in the spleens (n = 4). Data represented mean ± SD. Statistical significance was calculated via Student's t‐test or one‐way ANOVA with Dunnett's multiple comparison tests (b,c,e,f); ns means no significant difference. *p*‐value: ^*^ *p* <0.05, ^**^ *p* <0.01, ^***^ *p* <0.001, and ^****^ *p* <0.0001.

The metastatic nodules in the lungs after different treatments were assessed. The remarkable metastatic lesions could be found in all treated groups except a group of G5‐CHC‐R+US (**Figure** **7**a, upper), which showed much lower lung weights as well (Figure 7b). Extensive metastatic lesions with agminated nuclei were further confirmed microscopically (Figure 7a, lower). These results indicated the excellent capacity of the US‐driven sono‐nanovaccines in impeding tumor metastasis.

Similar to what was observed in the orthotopic pancreatic tumor model, G5‐CHC‐R+US treatment induced higher numbers of matured DCs in lymph nodes (Figure S42, Supporting Information) and more activated CD8^+^ T cells in the splenocytes (Figure 7c–e; Figure S43a, Supporting Information), indicating the initiation of the robust systemic immune responses. Furthermore, significantly increased effector/memory CD8^+^ T cells which express high levels of CD44 adhesion molecules, were observed in G5‐CHC‐R+US treated group (Figure 7f; Figure S43b, Supporting Information). These findings verified that the sono‐nanovaccines might be able to protect against tumor relapse by inducing durable immunological memory.

## 3 Discussion and Conclusion

High heterogeneity within and between cancer types, insufficient identification of tumor‐specific antigens, and unsatisfactory vaccine delivery efficiency emerge as major challenges lying in developing therapeutic cancer vaccines as well as their oncologic armamentarium expansions.^[^ ^5^, ^38^ ^]^ Apart from that, immunosuppressive TMEs of the majority of solid tumors further shield the efficaciousness of cancer vaccines.^[^ ^39^ ^]^ Herein, we designed a kind of in situ sono‐nanovaccines named G5‐CHC‐R, codelivery of the sonosentisizer, CHC, and the TLR7/8 agonist, R848. Under the free‐field based whole‐body US irradiation, G5‐CHC‐R induced cytotoxic effects toward tumor cells, allowing abundant release of immunogenic compounds and tumor‐associated antigens, which triggered DCs activation, cross‐presentation, and priming adaptive immune responses. The “cold” TME started to be warmed up. Concurrently, the NRT‐triggered release of R848 in hypoxia at tumor sites reinforced the complete converting of TME from “warm to hot” via phenotypic transforming of TAMs and down‐regulating MDSCs, ultimately facilitating cytotoxic effector T cells functioning locally and systemically. The free‐field based whole‐body US‐driven sono‐nanovaccines, G5‐CHC‐R could fulfill the sono‐immunotherapeutic requirements for various tumors with different malignancies, which had been demonstrated across orthotopic pancreatic cancer with intestinal metastasis and breast cancer with lung metastasis.

The excellent tumor penetration and retention of the sono‐nanovaccines could be addressed from two aspects. On one hand, size is one key parameter affecting the tumor accumulation capacity of nanocarriers.^[^ ^40^ ^]^ For example, Kataoka et al^[^ ^41^ ^]^ proposed that compared to the large nanoparticles (e.g., 50, 70, and 100 nm), only small ones (<30 nm) could pass through poorly permeable pancreatic cancer stroma, suggesting the size‐dependency of nanoparticles in tumor penetrating. The nano‐scaffold used in our study was highly branched poly(amidoamine) (G5 PAMAM) dendrimers with super small size (≈6 nm). After three steps of conjugation, the size of the resultant G5‐CHC‐R was increased to ≈12 nm. The desirable property in particle size largely benefited its penetration and accumulation toward tumors. On the other hand, there have been many studies suggesting that the porphyrin‐type molecules were prone to binding with the low‐density lipoproteins (LDL) in the blood circulation,^[^ ^42^ ^]^ and further being internalized by low‐density lipoprotein receptor‐related protein (LRP)‐mediated endocytosis.^[^ ^42^, ^43^ ^]^ LRP is highly expressed on the surfaces of tumor cells.^[^ ^44^ ^]^ Therefore, CHC as a porphyrinic derivative, being covalently modified on top of G5, could be more favorable for the improvement of tumor specificity of the sono‐nanovaccines.

The process of ROS generation consumes oxygen, which may drive solid tumors into higher hypoxic status, thus limiting the efficacy of ROS‐oriented therapies, such as photodynamic therapy (PDT).^[^ ^45^ ^]^ Emerging strategies have been applied to improve local oxygenation via hyperbaric oxygen therapy^[^ ^46^ ^]^ and in situ generation of oxygen.^[^ ^47^ ^]^ Instead, ultrasonic treatment might be able to alleviate the state of cell hypoxia by modifying the permeability of cell membrane, promoting metabolism as well as accelerating blood and lymphatic circulation in tumor situ.^[^ ^48^ ^]^ Consistently in our study, the significantly decreased HIF‐1α expression in tumor tissues was observed in both G5‐CHC+US and G5‐CHC‐R+US treated mice (Figure 3i), reflecting the improved tumor oxygenation by whole‐body SDT process. In addition, ROS has been reported to down‐regulate the activity of HIF‐1α via inhibiting HIF‐1 DNA‐binding activity under hypoxia.^[^ ^9c^ ^]^ The down‐regulating of HIF‐1α expression in both G5‐CHC+US and G5‐CHC‐R+US treated groups revealed an association with enhanced ROS levels via facilitated delivery of CHC to tumors by G5 platform. Given the outstanding tumor specificity, the sono‐nanovaccines were selectively accumulated in tumor tissues in vivo (Figure 3c,d; Figure S27, Supporting Information), resulting in the abundant ROS generation in the tumor area under US irradiation. The cellular damages were observed neither in para‐carcinoma tissues (Figure 7a) nor in other major organs (Figure S31, Supporting Information), suggesting negligible toxicity toward normal tissues/cells.

So far, the studies regarding to SDT‐based cancer therapy have been mostly conducted by using a low‐intensity focused ultrasound probe, which performs US in a singlet transducer focusing on a small region of the tumor.^[^ ^49^ ^]^ To enlarge the focusing range, the multiple phased array transducer has been applied as well, however still being limited by the tumor positioning.^[^ ^49^, ^50^ ^]^ In the meantime, the current existing ultrasonic devices unavoidably produce the waves in a significant standing‐wave pattern owing to the reflection at the interfaces between water/culture medium/coupling agent and air.^[^ ^51^ ^]^ We previously established a free‐field based whole‐body US irradiation system via equipping five US transducers in one temperature‐controllable water‐bath,^[^ ^9a^ ^]^ wherein the mouse immersed in water in order to allow the US waves to evenly penetrate the bodies of small animals. Compared to standing‐wave ultrasound, free‐field ultrasound exhibits significant advantages of stable acoustic pressure amplitudes, less attenuation, and reflection as well as reduced mechanical damage to the normal tissues.^[^ ^9a^ ^]^ In this work, the free‐field based whole‐body US irradiation system was pioneered to combine with the sono‐nanovaccines, consequently leading to effective attenuation of tumor growth and metastasis. By applying free‐field‐based whole‐body US irradiation, it is more likely to target multiple tumor sites simultaneously and/or even circulation tumor clusters in vivo. Our proof‐of‐concept study may provide new insights for designing a more effective and comfortable SDT modality for clinical applications.

The free‐field based whole‐body US‐driven sono‐nanovaccines are offered as one of the optimal ways to construct in situ tumor vaccines to achieve personalized treatment across a broad of spectrum of cancer types, unlocking immeasurable potential for translational research in the clinic.

## 4 Experimental Section

### Materials

Carboxyl PEG_2k_‐N_3_ and Methoxyl PEG_2k_ carboxyl were purchased from Ponsure Biological. Sodium ascorbate, Tri[(1‐benzyl‐1H‐1,2,3‐triazol‐4‐yl) methyl] amine (TBTA), and CuI were purchased from Accela ChemBio Co., Ltd. (Shanghai, China). N‐(3‐Dimethylaminopropyl)‐*N*’‐ethylcarbodiimide hydrochloride (EDC), 1‐Hydroxybenzotriazole (HOBt), N‐Hydroxysuccinimide (NHS), 4‐Dimethylaminopyridine (DMAP), and N,N‐Diisopropylethylamine (DIPEA) were purchased from Energy Chemical (Shanghai, China). PAMAM 5.0 was purchased from Weihai CY Dendrimer Technology Co., Ltd. (Shandong, China). 3‐Hydroxy‐4‐nitrobenzaldehyde was purchased from Shanghai Haohong Biomedical Technology Co., Ltd. (Shanghai, China). Propargyl bromide was purchased from Macklin Biochemical Co., Ltd. 4‐Nitrophenyl chloroformate and DPBF from Sigma–Aldrich. Antifade Mounting Medium with DAPI was purchased from Beyotime Biotech. Inc. (Shanghai, China). Resiquimod (R848) was purchased from Jiangsu Aikon (Jiangsu, China). Detailed information on the antibodies such as fluorescent antibody type, manufacturer, clone, and catalog number used in this study was provided in Tables S1–S3 (Supporting Information).

### Characterizations

The absorption spectrum of nanoparticles was obtained using a Lambda UV–vis spectrophotometer (PerkinElmer, USA). Analysis of nanoparticle size and surface potential by the ZS90 Zeta Sizer (Malvern, UK).

### Drug Loading Capacity

The UV–vis (Lambda 750 S, PerkinElmer) method was employed to ascertain the concentrations of CHC and R848 in G5‐CHC‐R, G5‐CHC, and G5‐R solutions. Different concentrations of free CHC (0.5, 1, 2, 4, 6, 8, 10, 12, 14, 16, 18, and 20 µg mL^−1^) or R848 (0.01, 0.02, 0.04, 0.06, 0.08 mg mL^−1^) were dissolved in DMSO/ DI water 8:2 (V/V), and the UV spectra at CHC 665 nm and R848 325 nm was measured. The content of CHC and R848 in G5‐CHC‐R, G5‐CHC, and G5‐R solutions was calculated by drawing the standard absorption curves. The drug loading efficiency (DLE) of CHC or R848 in G5 was calculated as follows: DLE (wt.%) = (loaded drug weight) / (total drug weight) × 100%

### In Vitro Measurement of Singlet Oxygen

Electron spin resonance (ESR) was carried out for the production of singlet oxygen (^1^O_2_) by using a Bruker E500 electron paramagnetic spectrometer. 2,2,6,6‐Tetramethylpiperidine (TEMP) was used as the trapping agent and was divided into three groups, including the TEMP group (control), TEMP+US group, and G5‐CHC‐R+US group, followed by the US irradiated (1 MHz, 3 W cm^−2^) for 10 min.

### In Vitro Generation of ROS

G5‐CHC‐R (CHC 15 µg mL^−1^) was mixed with 50 µg mL^−1^ of DPBF (1 mL DMSO], followed by free‐field US irradiated (1 MHz, 3 W cm^−2^) for 10 min. Aliquots were taken at specific time points (0, 2, 4, 6, 8, and 10 min), and the absorbance change of DPBF at 407 nm was recorded via UV–vis (Cary 100, Agilent).

### In Vitro Release of R848

HPLC was used to analyze prodrug activation in vitro (Agilent 1260 infinity). First, G5‐CHC‐R (250 µL) and NADH (100 µM) were dissolved in PBS buffer with pH = 7.4. Then, nitrogen was employed to remove the solution's air. Following that, nitroreductase (100 µg/mL) was added, and the reaction mixture was incubated for 6 h at 37°C. The solution was purified by a 0.22 µm polyvinylidene fluoride filter. The HPLC gradient elution method was used with 70% methanol, a detection wavelength of 280 nm.

### Hemolysis Assay

The efibrotic sheep blood was washed with saline (10 mL) three times (1500 rpm, 15 min, 4 °C) and resuspended with saline to obtain 2% erythrocyte suspension. G5‐CHC‐R of different concentrations (32.15, 62.5, 125, 250, and 500 µg mL^−1^) was dissolved in saline and incubated with 2% erythrocyte solution at 37 °C for 1 h. The hemolytic reaction was terminated by an ice bath, after incubation at 37 °C for 1 h, the mixture was then centrifuged (800 g, 15 min, 4 °C).

### Animals

Female C57BL/6J mice aged 6 weeks and weighing 18–20 g were obtained from Liaoning Changsheng Biotechnology (Benxi, China). Female BALB/c mice aged 6 weeks and weighing 18–20 g were obtained from Liaoning Changsheng Biotechnology (Benxi, China). Animals were housed in standard housing at a temperature of 24 °C and on a 12 h light/12 h dark cycle. All animal experimental procedures employed in this study were approved by the Biological and Medical Ethics Committee (BMEC) of Dalian University of Technology (DUTSCE230802‐01).

### Cell Culture

Pan02 murine pancreatic cancer cells were supplied by the National Cell Line Resource Infrastructure (Beijing, China). These Pan02 cells were incubated in a cell culture plate in Dulbecco's modified Eagle's medium (DMEM, HyClone) supplemented with 5% heat‐inactivated fetal bovine serum (PAN‐Seratech), 1% penicillin‐streptomycin (HyClone) at 37 °C in a humidified, 5% CO_2_ atmosphere. iCell Bioscience Inc. (Shanghai, China) supplied the 4T1 murine breast cancer cells. These 4T1 cells were incubated in a cell culture plate in RPMI‐1640 (HyClone) supplemented with 10% heat‐inactivated fetal bovine serum (PAN‐Seratech), 1% penicillin‐streptomycin (HyClone) at 37 °C under a humidified, 5% CO_2_ atmosphere.

### Cellar Uptake

4T1 and Pan02 cells (1 × 10^5^ cells) were seeded in a cell climbing film (20 mm Diameter) and then cultured for 24 h. Following this, the cells were incubated with fresh medium containing G5‐CHC‐R (CHC 50 µg mL^−1^). Subsequently, the cells were cultured for 1, 4, 8, and 12 h. These treated cells were washed three times with PBS, fixed with 4% paraformaldehyde (30 min, 4 °C), and stained with DAPI for 20 min at room temperature. Imaging was performed using a Leica DMi8 microscope and the THUNDER Imaging System.

### Detailed Protocols for 3D Tumor Spheroids

In preparation of a 1% agarose solution, 10 mL of DMEM (High glucose) medium was mixed with 0.1 g of agarose. The agarose solution was brought to a temperature above 70 °C before aliquoting 100 µL into each well of a 96‐well plate, and the plate was incubated at 37 °C for at least 1 h. The cells were harvested using trypsin, and utilized a hemocytometer to determine the cell concentration in the suspension and diluted the cell suspension to 10000 cells mL^−1^ and added 100 µL to the well containing the cured agarose bed. The cells in an incubator set to 37 °C and 5% CO_2_. On the 7th day, spheroids were incubated for 4 h with free CHC and G5‐CHC‐R (equal quantities of CHC 50 µg mL^−1^) at 37 °C under 5% CO_2_. Confocal fluorescence microscopy (FV1000, Japan) was used to visualize 3D tumor spheroids.

### In Vitro Sonocytotoxicity Assay

The cells were seeded in cylindrical polystyrene tissue culture tubes (2 × 10^5^ cells) and incubated at 37 °C for 12 h in a complete medium. All tubes were randomly divided into the G5‐CHC group, the G5‐CHC + US group, the G5‐CHC‐R, and the G5‐CHC‐R+ US group. DMEM medium was used to dilute drugs to varying concentrations (0, 12.5, 25, 50, 100, and 200 µg mL^−1^). The drugs were then added to the cells and were incubated in the dark for 4 h and washed 2 times using PBS. After resuspending cells in 1 ml of DMEM medium (serum‐free), they were treated with free‐field US (1 MHz, 3 W cm^−2^, 20 min). The cells were incubated at 37 °C for 4 h, Afterward, each group was added 1 mg mL^−1^ MTT reagent (Yeasen), and the tube was kept at 37 °C for 4 h. After removing the medium, each tube was supplemented with 100 µL DMSO and shaken for 10 min. Each tube's absorbance (OD) at 490 nm was determined using a microplate reader (spark). The absorbance of blank cells (OD control) was used as a control, and the cell visibility percent was computed by dividing the OD value by the OD value of the control.

### Detection of Cellular ROS Generation and Mitochondrial Damage

4T1 cells were seeded at a density of 1 × 10^6^ cells into cylindrical polystyrene tissue culture tubes. After 12 h, the medium was replaced with a fresh medium containing G5‐CHC‐R (CHC 100 µg mL^−1^) for 4 h at 37 °C under 5% CO_2_. Afterward, the cells were incubated with 20 µm DCFH‐DA (Sigma‐Aldrich) for 30 min. Following that, cells were washed three times with PBS and treated with free‐field US irradiated (1 MHz, 3 W cm^−2^, 20 min). The mitochondrial damage was determined according to the manufacturer's instructions using a Mitochondrial Membrane Potential Assay Kit with Rhodamine 123 (Beyotime Biotechnology). Imaging was performed using a Leica DMi8 microscope and the THUNDER Imaging System.

### Isolation of Bone Marrow‐Derived Macrophages (BMDM)

BMDMs were isolated from C57BL/6 mice femurs and tibiae. The femur and tibia were cut with scissors to expose the marrow cavity, and the bone marrow cells were flushed out via injecting RPMI 1640 culture medium. The collected bone marrow cells were filtered via a 40 µm cell strainer, followed by centrifugation at 1800 rpm for 5 min, the cells were suspended in red cells lysis buffer (Beyotime Biotechnology) for 2 min. Afterward, the cells were centrifuged and resuspended in RPMI 1640 medium. The bone marrow cells were seeded on a 24‐well culture plate (each well contained 1 × 10^5^ cells) in RPMI‐1640 (HyClone) supplemented with 10% heat‐inactivated fetal bovine serum (PAN‐Seratech), 1% penicillin‐streptomycin (HyClone), 5% Sodium Pyruvate (100 nm, gibco) and 20 ng mL^−1^ recombinant murine M‐CSF (Biolegend). After seven days of incubation (medium was replaced every two days), BMDM were stimulated into M2 macrophages using IL‐4 (20 ng mL^−1^) treatment for 48 h.

### M2 Macrophage Repolarization

M2 Macrophages were incubated with free R848 (2 µg mL^−1^), NADH (100 µM, Aladdin), G5‐R (30 µg mL^−1^), G5‐ R (30 µg mL^−1^)+NTR (100 µg mL^−1^, Sigma)+NADH (100 µM, Aladdin). Following treatment for 24 h, the cells were collected, and M1‐related markers (F4/80^+^CD86^+^CD206^−^) and M2‐related markers (F4/80^+^CD86^−^CD206^+^) were detected by flow cytometry using an Attune NxT device (Thermo Fisher Scientific).

### CRT Exposure and ATP Release

The western blot was used to analyze CRT exposure. The 4T1 cells were resuspended at a density of 6 × 10^5^ in serum‐free media. Free CHC, G5‐CHC, G5‐CHC‐R (CHC 100 µg mL^−1^), and G5‐R (30 µg mL^−1^) were used to treat the cells. The cells were incubated in the dark for 4 h and washed twice with PBS. Then free‐field US irradiated (1 MHz, 3 W cm^−2^, 20 min), the cells were placed in the dark for 4 h. 4T1 cells in each group were harvested and 100 µL of RIPA buffer (Solarbio) was added to each well and rocked for 5 min on ice. Electrophoresis was employed to separate the total proteins and the separated proteins were transferred onto a PVDF membrane (Millipore, Billerica, MA, USA). The PVDF membrane was blocked with 5% skim milk in TBST (Tris‐buffered saline with 0.05%Tween 20) for 1 h at room temperature. After washing three times using TBST, the PVDF membranes were incubated in a buffer containing primary antibodies CRT and β‐actin (1:1000, Cell Signaling Technology, #12 238) and allowed to bind overnight at 4 °C. After washing three times using TBST, the membrane was incubated using an HRP‐labeled Goat anti‐Rabbit secondary antibody (1:2000; Proteintech) for 2 h at room temperature, then imaged with an ECL substrate (Bio‐Rad, USA). Enhanced ATP Assay Kit (Beyotime Biotechnology, S0027) was used as directed by the manufacturer to measure the ATP levels in the cell culture medium.

### Biodistribution, Penetration, and In Vivo Imaging

The biodistribution‐free CHC (15 mg kg^−1^ CHC), G5‐CHC (15 mg kg^−1^ CHC), and G5‐CHC‐R (15 mg kg^−1^ CHC) on mice bearing 4T1 tumor in the second pair of mammary glands of BALB/c mice and the orthotopic pancreatic cancer model were investigated. The fluorescence imaging was conducted at 1, 4, 8, and 12 h and sacrificed at 12 h. The heart, liver, spleen, kidney, and tumor were harvested and imaged in vivo by a NightOWL II LB983 small animal (Excitation wavelength: 665 nm, Emission wavelength: 700 nm).

### Biodistribution

The tumor‐bearing mice were injected with G5‐CHC‐R (15 mg kg^−1^ CHC) via the tail vein. Mice were executed at different time points (12, 24, 48, and 72 h) and major organs and tumors were taken for fluorescence imaging.

### An Orthotopic Pancreatic Cancer Model

Pan02 cells were injected into the pancreas to establish an orthotopic pancreatic cancer model. Pan02 cells were trypsinized and resuspended in a 1:1 PBS/Matrigel solution (Corning). Female C57BL/6J mice (six weeks old, 17 g) were anesthetized with Avertin (240 mg kg^−1^) via intraperitoneal injection. A 1 cm incision was made in the peritoneum below the sternum in the midabdominal region. This was accomplished by injecting 5 × 10^5^ Pan02 cells (25 µL) into the pancreas tail. A two‐layer suture with 4‐0 chromic catgut was used to close the abdomen, and antibiotic ointment was applied. Five days later, pancreatic cancer treatment. Mice were randomly assigned to one of five groups (n = 5) and intravenously injected with PBS, free CHC, G5‐CHC, G5‐R, and G5‐CHC‐R at 15 mg CHC kg^−1^ body weight (for G5‐R, 1 mg R848 kg^−1^ body weight). The pancreatic tumors were treated with free‐field US irradiated (1.0 MHz, 2 W cm^−2^, 20 min) 4 h after I.V. injection of free CHC + US, G5‐CHC + US, and G5‐CHC‐R + US. All mice were kept away from light throughout the experiment.

On day 24 after different treatments, micro‐PET/CT measurements were performed to assess tumor progression. All scans were conducted on a Super Nova PET/CT scanner for small animals (PINGSENG Healthcare, Jiangsu, China). Only water was provided for the orthotopic pancreatic cancer model that would be monitored via ^18^F‐FAPI micro‐PET/CT after a 24‐h fast. The body weights of the mice were determined, and they were anesthetized with Avertin before receiving a tail vein injection of 5.5–7.4 Mbq/0.1 mL ^18^F‐FAPI. In this region, CT scans of the abdomen were performed. PET data were collected for 15 min after a delay of 30 min to allow for ^18^F‐FAPI uptake.

### Murine Breast Cancer Model with Lung Metastases

To establish a model of breast cancer with spontaneous lung metastasis, 5 × 10^5^ 4T1 cells suspended (50 µL) in PBS were injected into the second pair of mammary glands of BALB/c mice. Five days later, the tumor volume had increased to 30 mm^3^ for treatment. 4T1 tumor‐bearing mice were divided into five groups (n = 5) and intravenously injected with PBS, free CHC, G5‐CHC, G5‐R, and G5‐CHC‐R at 15 mg CHC kg^−1^ body weight (for G5‐R, 1 mg R848 kg^−1^ body weight). The tumor volume (V) was calculated using the formula V = L W^2^/2, where L and W denote the tumor's length and width, respectively (tumor volume was measured at a one‐day interval). At the conclusion of the experiment, mice were sacrificed, and their breasts were removed, weighed, photographed, and analyzed pathologically. Finally, the lung was harvested and fixed using Bouin's solution.

### Flow Cytometric Analysis of the TME

At the end of treatment, pancreatic cancer tumor tissues were minced into small pieces with scissors and digested in DMEM containing 0.5 mg mL^−1^ Collagenase IV (Yeasen), 0.5 mg mL^−1^ Collagenase I (Yeasen), and 25 µg mL^−1^ DNAse 1. (Aladdin). After 1 h of incubation at 37 °C, samples were ground and filtered through 70 µm cell strainers, then centrifuged at 1800 rpm for 5 min at 4 °C. Orthotopic 4T1 tumors were minced into small pieces with scissors and digested in DMEM containing 0.5 mg mL^−1^ Collagenase IV (Yeasen) and 0.5 mg mL^−1^ Collagenase I (Yeasen). The samples were incubated at 37 °C for 1 h 30 min, samples were ground and filtered through 70 µm cell strainers, then centrifuged at 1800 rpm for 5 min at 4 °C. The collected single‐cell suspensions were stained with fluorescence‐labeled antibodies. Flow cytometry using an Attune NxT device (Thermo Fisher Scientific) and analyzed by FlowJo software 10. Fluorescence‐conjugated antibodies used in flow cytometry were summarized in Table S1 (Supporting Information).

### Flow Cytometric Analysis of the Lymph Nodes and Spleens

At the end of the experiment, Following the established protocol.^[^ ^52^ ^]^ lymph nodes and spleens were harvested from mice and prepared into single‐cell suspensions. Flow cytometry was stained immediately on the single‐cell suspension (Thermo Fisher Scientific, Attune NxT). Fluorescence‐conjugated anti‐mouse antibodies and reagents to label cells could be found in Table S1 (Supporting Information).

### Immunofluorescence Analyses

Tumors were harvested, fixed in Tissue‐Tek (OCT compound, Servicebio), and kept frozen at −80 °C. Then, tissues were cryo‐sectioned (thickness, 10 µm), fixed in 4% paraformaldehyde at room temperature for 30 min, and rinsed with PBS three times. Afterward, the samples were permeabilized with Enhanced Immunostaining Permeabilization Solution (Beyotime Biotechnology) for 30 min and rinsed thrice with PBS, then blocked with Immunol Staining Blocking Buffer (Beyotime Biotechnology) for 1 h and washed thrice with PBS. The samples were incubated with primary antibodies overnight at 4 °C. The sections were rinsed three times with PBS, incubated for 1 h in the dark with secondary antibodies, washed three times with PBS, and stained with DAPI (4′,6‐diamidino‐2‐phenylindole). Imaging was performed using a Leica DMi8 microscope and the THUNDER Imaging System. Tables S2 and S3 (Supporting Information) detail the primary and secondary antibodies.

### Statistical Analysis

GraphPad Prism 8 software was used to conduct the statistical analysis. The student's *t*‐test was used to compare the two groups. Multiple group comparisons were conducted using one‐way analysis of variance (ANOVA). Asterisks represent different levels of significance: ^*^ *p* <0.05, ^**^ *p* <0.01, ^***^ *p* <0.001, and ^****^ *p* <0.0001; *ns*, not significant. FlowJo 10 was used to perform all the flow cytometry analyses.

## Conflict of Interest

The authors declare no conflict of interest.

## Supporting information

Supporting Information

## Data Availability

The data that support the findings of this study are available from the corresponding author upon reasonable request.
