# Supplementary material for: Spatiotemporal Controllable Sono‐Nanovaccines Driven by Free‐Field Based Whole‐Body Ultrasound for Personalized Cancer Therapy
Source: Adv Sci (Weinh). 2024 Feb 2;11(14):2307920. doi: 10.1002/advs.202307920 (PMC11005707; doi:10.1002/advs.202307920)
Supplement: Supplementary file 1 — Supporting Information [file ADVS-11-2307920-s001.pdf]

ADVANCED SCIENCE
Open Access

Supporting Information

for *Adv. Sci.*, DOI 10.1002/advs.202307920

Spatiotemporal Controllable Sono-Nanovaccines Driven by Free-Field Based Whole-Body Ultrasound for Personalized Cancer Therapy

Yang Wang, Guangzhe Li, Jianlong Su, Yiming Liu, Xiaomai Zhang, Guanyi Zhang, Zhihao Wu, Jinrong Li, Yuxuan Zhang, Xu Wang, Zejia Yang, Ruimin Wang, Chengdong Wang, Liu Wang, Fangfang Sun, Weijie Zhao, Xuejian Wang, Xiaojun Peng and Kun Shao**

Supporting Information

Spatiotemporal controllable sono-nanovaccines driven by free-field based whole-body ultrasound for personalized cancer therapy

Yang Wang[1], *Guangzhe Li*[2]*, *Jianlong Su*[1], *Yiming Liu*[1], *Xiaomai Zhang*[1], *Guanyi Zhang*[1], *Zhihao Wu*[1], *Jinrong Li*[1], *Yuxuan Zhang*[1], *Xu Wang*[1], *Zejia Yang*[1], *Ruimin Wang*[1], *Chengdong Wang*[3], *Liu Wang*[2], *Fangfang Sun*[3], *Weijie Zhao*[2], *Xuejian Wang*[4], *Xiaojun Peng*[1], *Kun Shao*[1]*

[1] State Key Laboratory of Fine Chemicals, School of Chemical Engineering, Dalian University of Technology, Dalian 116024, China.

[2] State Key Laboratory of Fine Chemicals, Department of Pharmacy, School of Chemical Engineering, Dalian University of Technology, Dalian 116024, China.

[3] Nuclear Medicine, First Affiliated Hospital of Dalian Medical University, Dalian 116021, China.

[4] Department of Urology, First Affiliated Hospital of Dalian Medical University, Dalian 116021, China.

*Corresponding authors. Email: liguangzhe@dlut.edu.cn (G. L.); shaok@dlut.edu.cn (K. S.);

1. Synthesis and Characterization

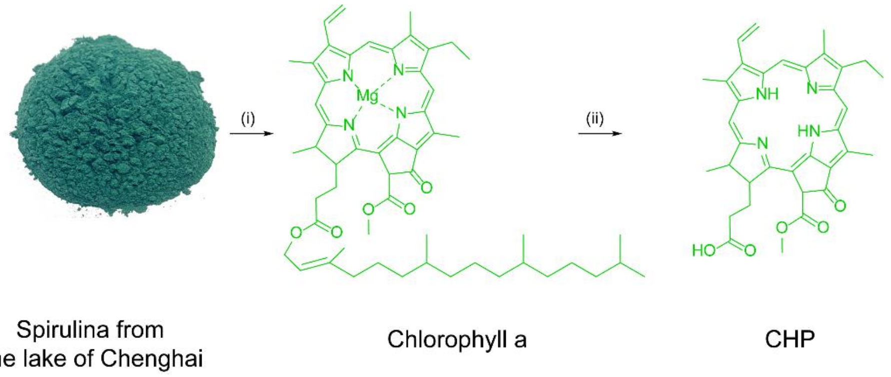

Figure S1. Structure and synthesis of Chenghai Pheophorbide a (CHP). Reagents and conditions: (i) Acetone, Reflux, 2 h; (ii) Et_2O, HCl, 1.1%.

1.1 Extraction of chlorophyll a

One hundred grams of spirulina platensis powders originated from Chenghai Lake were soaked in 500 mL of acetone, and transferred into a 1 L three-necked flask. The three-necked flask was placed in an oil bath equipped with an electrical stirring device and a reflux condenser. The oil bath was heated to 65°C. Once the solvent in the three-necked flask reached a temperature of 56°C, it started refluxing. Kept the reflux for 2 h. Subsequently, stopped heating and kept stirring until the solution in the three-necked flask naturally cooled down to 30°C. Vacuum filtration was used for solid liquid separation. The residue was washed with acetone until the filtrate exhibited a light colour. The afore-mentioned extraction steps were repeated thrice, and the resulting filtrates were combined and subjected to rotary evaporation to obtain a spirulina-derived chlorophyll extract.

1.2 Preparation of Chenghai Pheophorbide a (CHP)

The spirulina-derived chlorophyll extract was dissolved in 300 mL of ethyl ether and transferred into a 1 L three-necked flask. Installed with a mechanical stirring device and drip funnel, nitrogen gas was introduced, and stirring was started while the mixture was gradually cooled down to -10°C in an ice-salt bath. Added 12 M HCl (150 mL) to the solution slowly at -10°C. Controlled the rate of adding HCl to maintain the temperature of the reaction mixture below 0°C. The resultant mixture was then warmed up to room temperature and stirred at the same temperature until chlorophyll a was consumed (TLC analysis, petroleum ether/ethyl acetate = 3:1). The reaction mixture was transferred into a 5 L separatory funnel and extracted thrice with petroleum ether (350 mL). The water layer was collected.

A saturated Na_2CO_3 solution was added dropwise to the collected solution to adjust pH to 4.0. The solid precipitate in dark green was separated via vacuum filtration using a Büchner funnel and washed with 1 vol.% aqueous propanoic acid solution. Subsequently, the obtained solid precipitate was placed in a vacuum for drying at 25°C. The dried solid was dissolved in dichloromethane (DCM) mixed with methanol in a ratio of 10:1 and transferred into an eggplant flask. When the solvent was removed via rotary evaporation, Chenghai Pheophorbide a (CHP, 1.08 g) was obtained (total yield, 1.1%).

A small amount of the magnesium-free chlorophyllin a was placed in an eppendorf tube and dissolved in chromatography-grade methanol (1.5 mL). The solution was drawn into a 2 mL syringe, filtered through a 0.22 μm membrane filter, and injected into a liquid sample vial. Subsequently, the purity of the compound was measured using a high-performance liquid chromatography (HPLC) system (Waters Alliance e2695 HPLC) equipped with a 2489 ultraviolet/visible detector. Methanol and ammonium acetate buffered salt solution, 100 mM were used as the mobile phase (detection wavelength, 400 nm; flow rate of the mobile phase, 1 mL min^{-1}; separation column, C18 reversed-phase column; injection volume, 20 μL). The purity of Chenghai Pheophorbide a (CHP) was 70.8%.

1.3 Synthesis of Chenghai chlorin (CHC)

CHC was synthesized through a two-step process using CHP as the raw material.

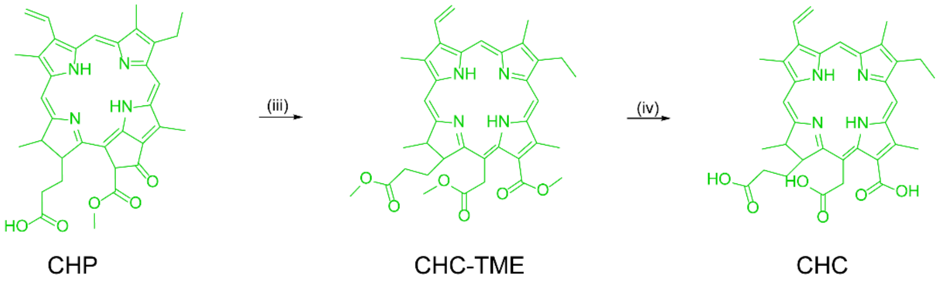

Figure S2. Structure and synthesis of CHC. Reagents and conditions: (iii) 5% H_2SO_4 in CH_3OH, 25 °C, 4 h; CH_3OH, CH_3ONa, 25 °C, 12 h; 23%; (iv) 5% KOH, THF, 40 °C, 12 h, 81%.

Step one: The extracted CHP (500 mg) was accurately weighed and thoroughly dissolved in 40 mL of 5% sulphuric acid in methanol. Subsequently, the solution was transferred into a 250 mL eggplant flask and stirred at 25°C for 4 h under nitrogen. The reaction progress was monitored *via* thin-layer chromatography (TLC). After the reaction was completed, the solvent was removed *via* rotary evaporation, and the residue was transferred into a separatory funnel. Extraction was performed thrice with deionized water (20 mL) and once with a saturated aqueous NaCl solution. The lower-layer solution was collected and dried over with anhydrous

Na_2SO_4. Subsequently, the solvent was removed *via* rotary evaporation to obtain the carboxyl methyl esterified product of CHP.

The carboxyl methyl esterified product of CHP was dissolved in methanol (25 mL) followed by the addition of sodium methoxide (1.5 mL). The obtained solution was transferred into a 50 mL eggplant flask and reacted at 25°C for 12 h under nitrogen. Reaction progress was monitored using TLC. Upon the reaction completion, formic acid (0.3 mL) was added to the system, and the solvent was removed *via* rotary evaporation. After dissolution in DCM, the solution was transferred into a separatory funnel. Extraction was performed thrice with deionized water and once with a saturated aqueous NaCl solution. The organic layer solution was collected, and dried over with anhydrous Na_2SO_4. Finally, the rotary evaporation of the solvent and column chromatographic separation were performed to obtain 117 mg of the product. ^1H NMR (400 MHz, $CDCl_3$) δ 9.68 (s, 1H), 9.54 (s, 1H), 8.73 (s, 1H), 8.04 (dd, J = 17.8, 11.5 Hz, 1H), 6.33 (d, J = 17.8 Hz, 1H), 6.12 (d, J = 11.5 Hz, 1H), 5.35 (d, J = 18.8 Hz, 1H), 5.28-5.20 (m, 1H), 4.41 (dd, J = 16.1, 8.5 Hz, 2H), 4.25 (s, 3H), 3.76 (s, 5H), 3.62 (s, 3H), 3.57 (s, 3H), 3.45 (s, 3H), 3.28 (s, 3H), 2.56 (d, J = 7.2 Hz, 1H), 2.20 (s, 2H), 1.75-1.68 (m, 7H), -1.31 (s, 1H), -1.47 (s, 1H). HRMS (ESI) *m/z:* $[M+H]^+$ calcd for $C_{37}H_{43}N_4O_6$ 639.3104; found, 639.3187.

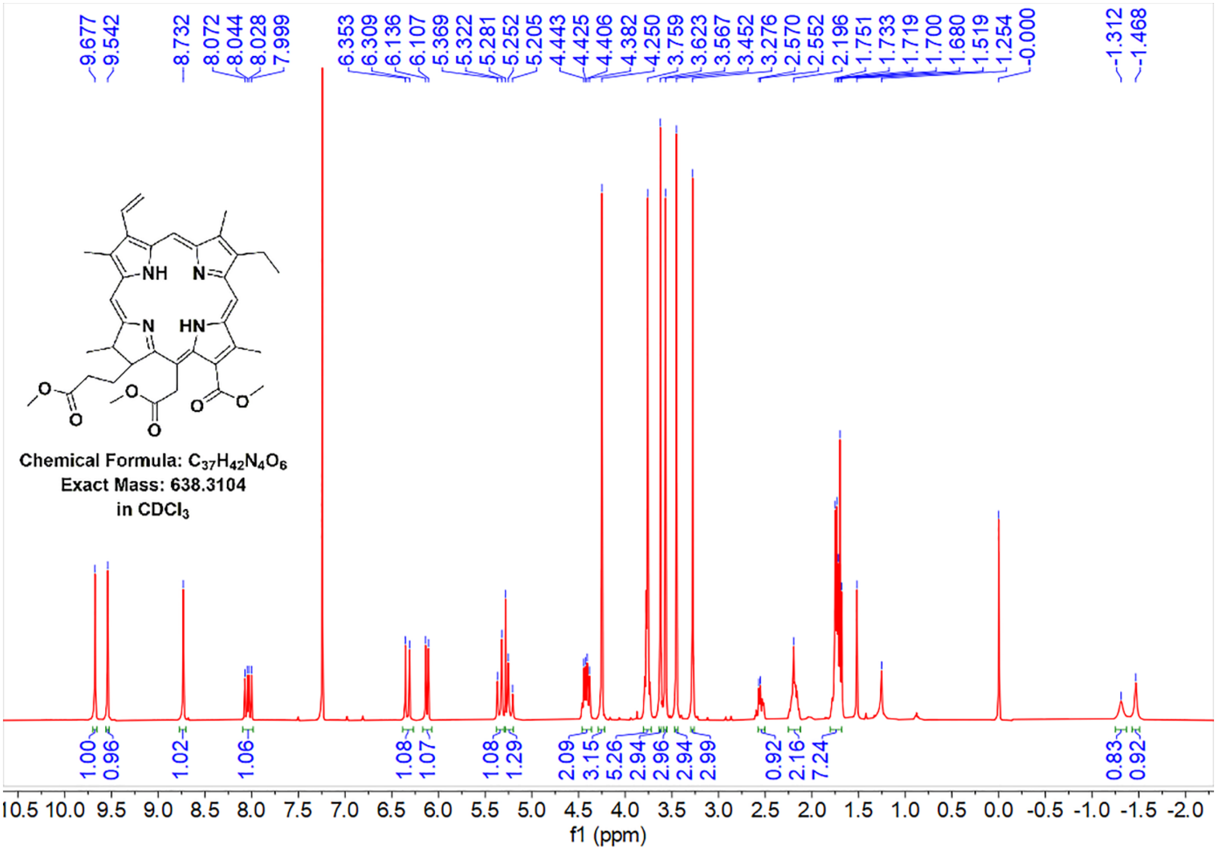

Figure S3. ^1H NMR spectrum of CHC-TME in $CDCl_3$

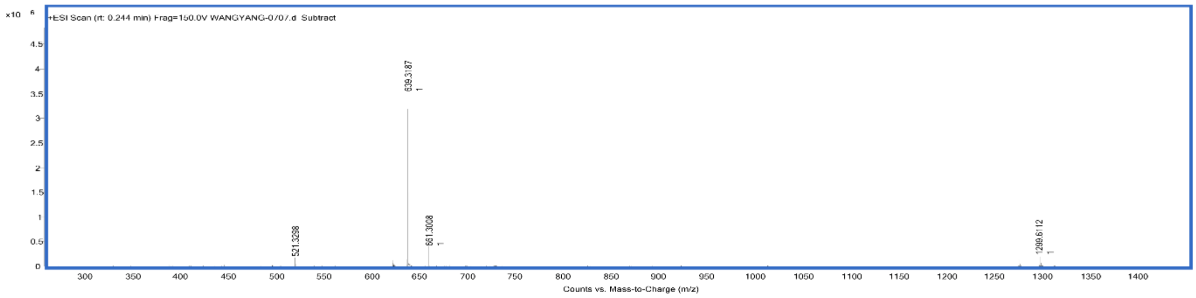

Figure S4. HRMS of CHC-TME.

Step two: To a solution of CHC-TME (45.5 mg) in THF, added 1 M KOH solution and refluxed reaction under nitrogen. Reaction progress was monitored *via* TLC. After 12 h of reaction, the organic solvent was removed *via* rotary evaporation. Thereafter, a 1 M aqueous hydrochloric acid solution was added to the reaction mixture to adjust its pH to approximately 4.0. The solid product was obtained *via* vacuum filtration using a Büchner funnel and washed with a 1 vol.% aqueous propanoic acid solution. Subsequently, the solid was dried in a vacuum drying oven and dissolved in a mixture of DCM and methanol with a 1:1 (v/v) ratio. After the solvent removal *via* rotary evaporation, 34.4 mg of CHC was ultimately obtained (yield: 81%).^{1}H NMR (400 MHz, DMSO-d_6) δ 9.74 (d, J = 28.2 Hz, 2H), 9.10 (s, 1H), 8.30 (dd, J = 17.9, 11.6 Hz, 1H), 6.45 (d, J = 16.3 Hz, 1H), 6.18 (d, J = 11.6 Hz, 1H), 5.42-5.28 (m, 2H), 4.61 (q, J = 7.1 Hz, 1H), 4.45 (d, J = 8.3 Hz, 1H), 3.80 (d, J = 7.7 Hz, 2H), 3.57 (s, 3H), 3.52 (s, 3H), 3.30 (s, 3H), 2.62 (dt, J = 16.4, 8.1 Hz, 1H), 2.32-2.25 (m, 1H), 2.10 (d, J = 13.2 Hz, 1H), 1.70-1.65 (m, 6H), 1.56-1.54 (m, 1H), -1.65 (s, 1H), -1.93 (s, 1H). HRMS (ES) *m/z:* [M-H] calcd for $C_{34}H_{35}N_4O_6$ 595.2635; found, 595.2576.

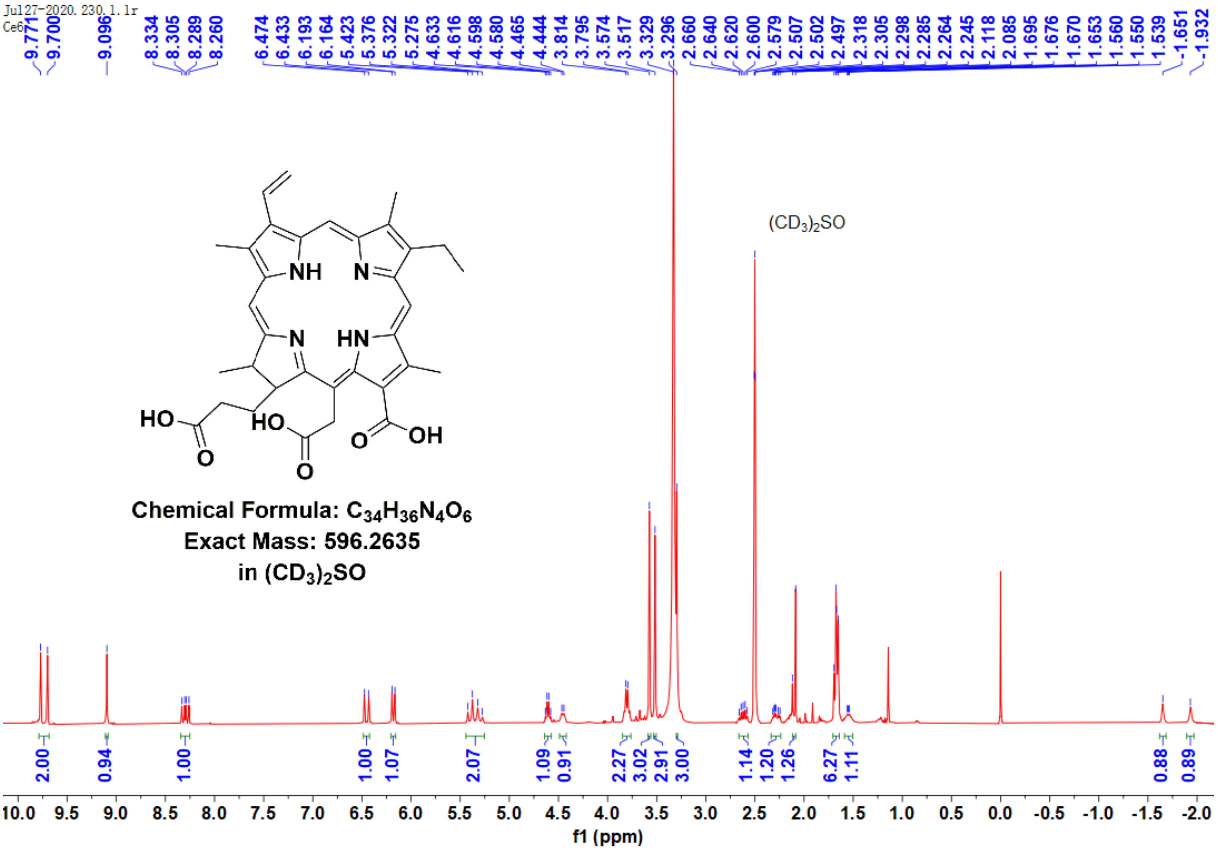

Figure S5. ^{1}H NMR spectrum of CHC in DMSO-d_6.

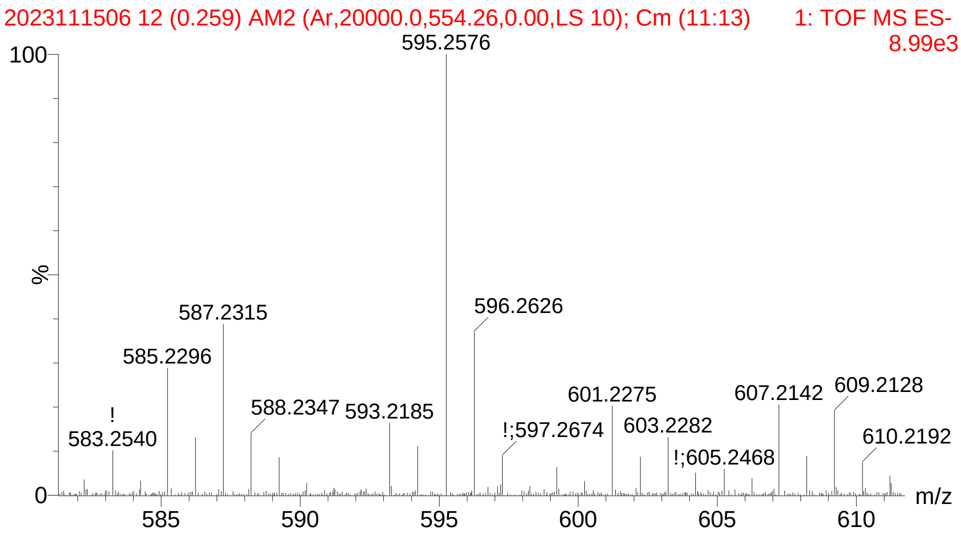

Figure S6. HRMS of CHC.

1.4 Structure and Synthesis of the R848-NTR-COOH

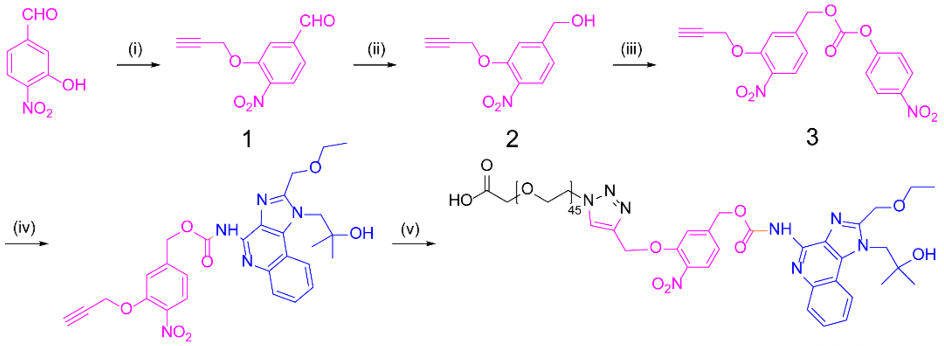

Figure. S7. Structure and synthesis of R848-NTR-COOH. Reagents and conditions: (i) Propargyl bromide, K_2CO_3, DMF, 50°C, 95%; (ii) NBH_4, MeOH; 0°C, 97%; (iii) 4-Nitrophenyl chloroformate, TEA, CH_2Cl_2, 0°C; (iv) R848, compound 3, TEA; (v) N_3-$(CH_2CH_2O)_n$-CH_2-COOH, TBTA, CuI, Sodium ascorbate.

Synthesis of the compound 1: A mixture of 3-hydroxy-4-nitrobenzaldehyde (0.5013 g, 3 mmol) and K_2CO_3 (0.800 g, 6 mmol) in DMF (10 mL) was mixed at room temperature for 30 min. Then, propargyl bromide (0.535 g, 4.5 mmol) was added, and the mixture was stirred at 60°C for 2 h. Following the assertion of the reaction's completion, the final mixture was cooled, filtered, and concentrated under a vacuum. The crude product was purified by a flash chromatography on silica gel (ethyl acetate/hexane = 3:10) to give a light-yellow solid of compound 1 (0.5852 g, 95% yield). ^{1}H NMR (400 MHz, $CDCl_3$) δ 10.07 (s, 1H), 7.95 (d, J = 8.1 Hz, 1H), 7.76 (d, J = 1.4 Hz, 1H), 7.61 (dd, J = 8.1, 1.5 Hz, 1H), 4.93 (d, J = 2.4 Hz, 2H), 2.63 (t, J = 2.4 Hz, 1H). HRMS (EI) *m/z:* calcd for $C_{10}H_7NO_4$ 205.0375; found, 205.0382.

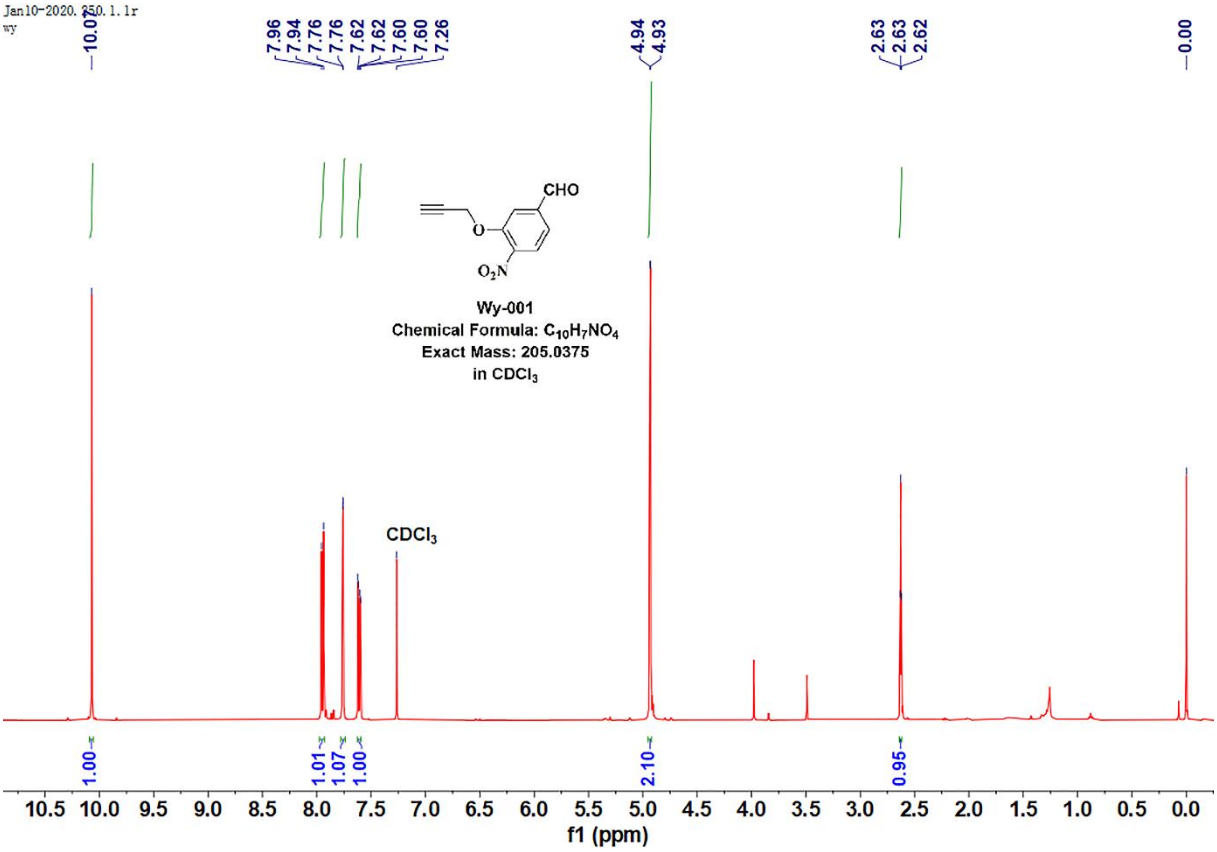

Figure S8. ^{1}H NMR spectrum of compound 1 in $CDCl_3$

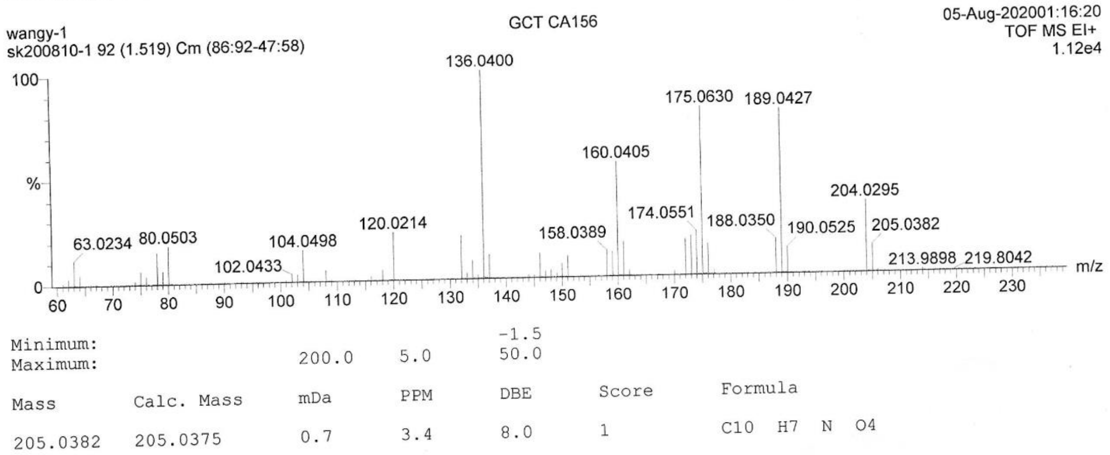

Page 1

Elemental Composition Report

Single Mass Analysis
Tolerance = 200.0 mDa / DBE: min = -1.5, max = 50.0
Isotope cluster parameters: Separation = 1.0 Abundance = 1.0%

Monoisotopic Mass, Odd and Even Electron Ions
8 formula(e) evaluated with 1 results within limits (up to 50 closest results for each mass)

Minimum:				-1.5		
Maximum:		200.0	5.0	50.0		
Mass	Calc. Mass	mDa	PPM	DBE	Score	Formula
205.0382	205.0375	0.7	3.4	8.0	1	C10 H7 N O4

Figure S9. HRMS of compound 1.

Synthesis of compound 2: Compound 2 (0.4114 g, 2.0 mmol) was dissolved in 10 mL of MeOH. $NaBH_4$ (0.1513 mg, 4 mmol) was added, and the reaction mixture was stirred in an ice bath while TLC was used to monitor the reaction. Following completion of the reaction, the solvent MeOH was removed using a rotary evaporator. Water (10 mL) was added and the mixture was extracted three times with ethyl acetate. Na_2SO_4 was used to dry the composite organic layer, and the solvent was removed using a rotary evaporator. The crude product was purified by a flash chromatography on silica gel (ethyl acetate:hexane = 1:1) to obtain a white solid of compound 2 (0.4012 g, 97% yield). ^{1}H NMR (400 MHz, $CDCl_3$) δ 7.87 (d, J = 8.3 Hz, 1H), 7.29 (s, 1H), 7.06-7.04 (m, 1H), 4.87 (d, J = 2.4 Hz, 2H), 4.80 (s, 2H), 2.59 (t, J = 2.4 Hz, 1H). HRMS (EI) *m/z:* calcd for $C_{10}H_9NO_4$ 207.0532; found, 207.0522.

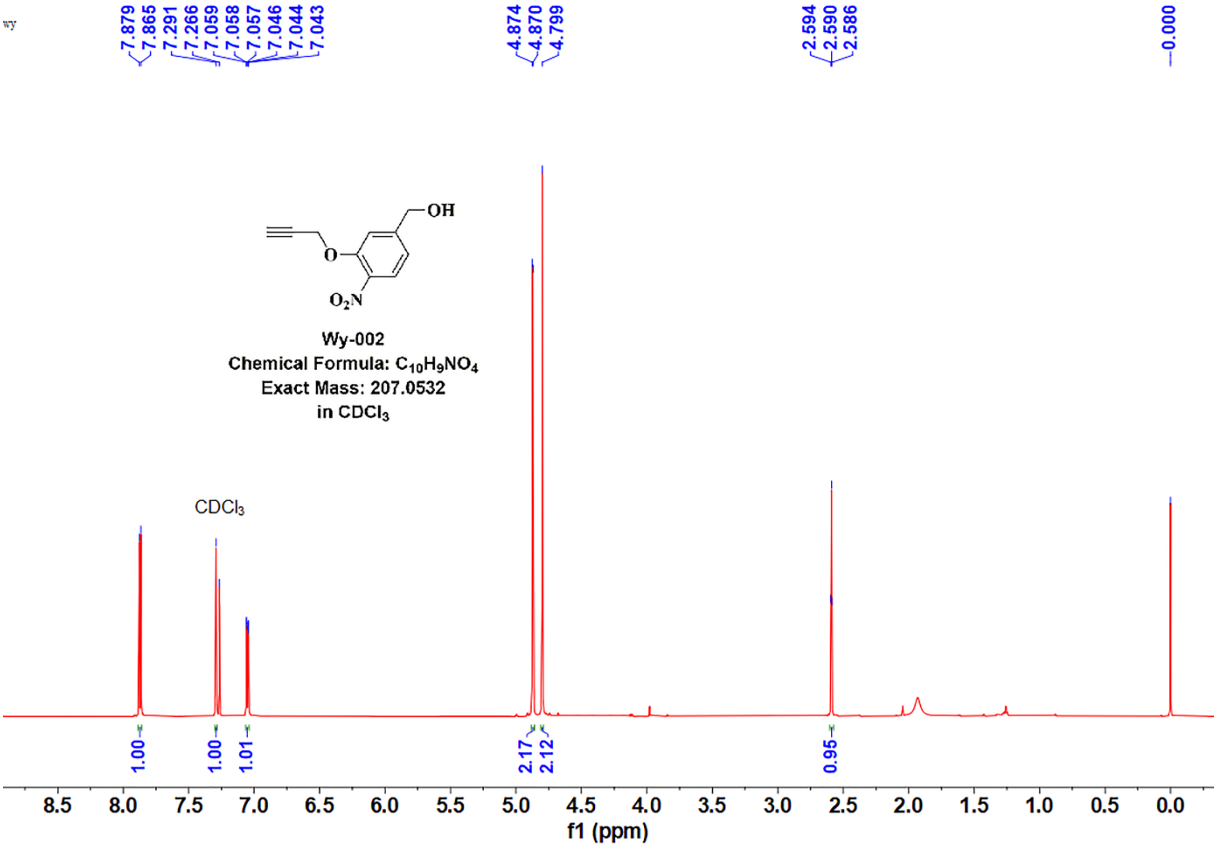

Figure S10. ^{1}H NMR spectrum of compound 2 in $CDCl_3$.

Elemental Composition Report Page 1

Single Mass Analysis
Tolerance = 200.0 mDa / DBE: min = -1.5, max = 50.0
Isotope cluster parameters: Separation = 1.0 Abundance = 1.0%

Monoisotopic Mass, Odd and Even Electron Ions
8 formula(e) evaluated with 1 results within limits (up to 50 closest results for each mass)

wangy-2 GCT CA156 05-Aug-202002:03:26
sk200810-2 271 (4.517) Cm (245:272-115:159) TOF MS EI+
4.05e4

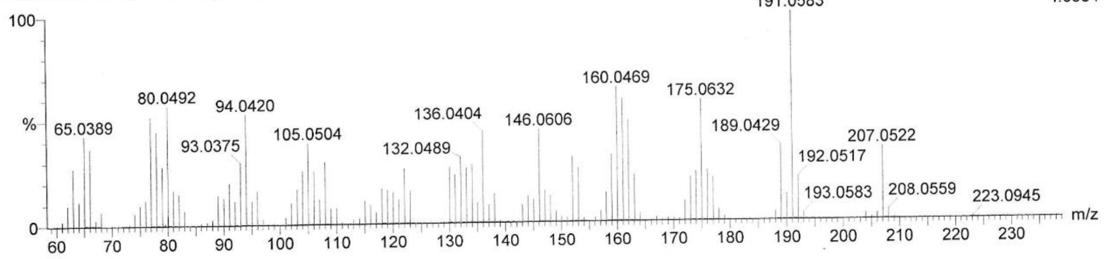

Minimum:				-1.5		
Maximum:		200.0	5.0	50.0		
Mass	Calc. Mass	mDa	PPM	DBE	Score	Formula
207.0522	207.0532	-1.0	-4.6	7.0	1	C10 H9 N O4

Figure S11. HRMS of compound 2.

Synthesis of compound 3: Compound 2 (0.0414 g, 0.2 mmol) was dissolved in DCM (4 mL). TFA was added (80 μL) and stirred for 30 minutes in an ice bath. To this solution was added 4-nitrophenyl chloroformate (0.0605 g, 0.3 mmol) and monitored using a TLC spot plate. Without further purification, the product of the reaction was used directly for the next step of synthesis.

Synthesis of compound 4: To a solution of Compound 3 in DCM (5 mL) added TEA (150 μL). To this solution was added R848 (0.0628 g) in DCM/DMF (4 mL /1 mL, V/V), and the mixture was stirred at room temperature for 12 h. The reaction was monitored using a TLC, and the solvent was evaporated using a rotary evaporator to yield a yellow oil. The crude product was purified by a flash chromatography on silica gel (dichloromethane:methanol = 50:1) to obtain a pale yellow solid (compound 4, 0.0828 g). ^{1}H NMR (400 MHz, $CDCl_3$) δ 8.16 (d, J = 8.3 Hz, 1H), 8.10 (d, J = 7.4 Hz, 1H), 7.88 (dd, J = 8.3, 2.0 Hz, 1H), 7.61 (t, J = 8.2 Hz, 1H), 7.49 (t, J = 7.7 Hz, 1H), 7.36 (s, 1H), 7.21 (d, J = 8.3 Hz, 1H), 5.36 (d, J = 2.1 Hz, 2H), 4.97-4.74 (m, 6H), 3.65 (qd, J = 6.9, 2.9 Hz, 2H), 2.62 (t, J = 2.3 Hz, 1H), 1.33 (s, 6H), 1.24 (dd, J = 7.0, 2.1 Hz, 3H). HRMS (ESI) *m/z:* $[M+H]^+$ calcd for $C_{28}H_{30}N_5O_7$, 548.2067; found, 548.2131.

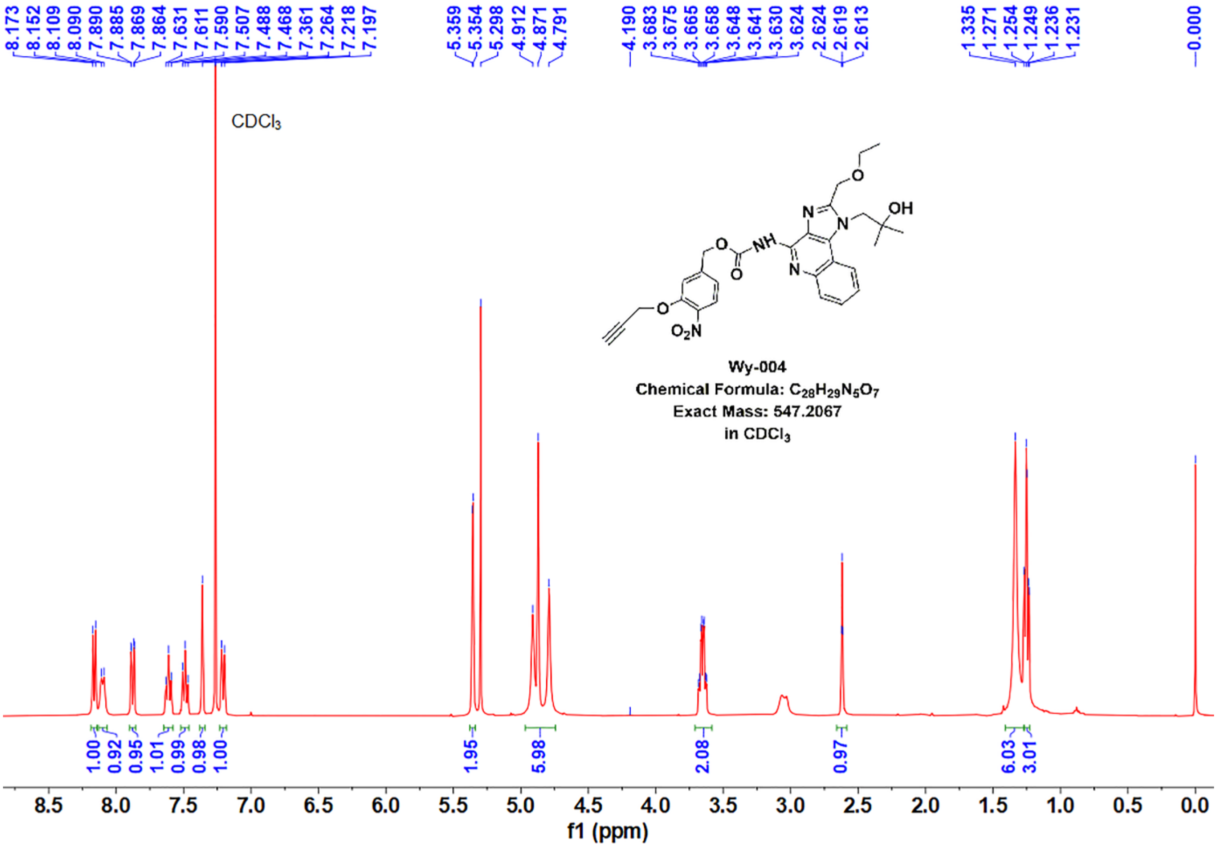

Figure S12. ^{1}H NMR spectrum of compound 4 in $CDCl_3$.

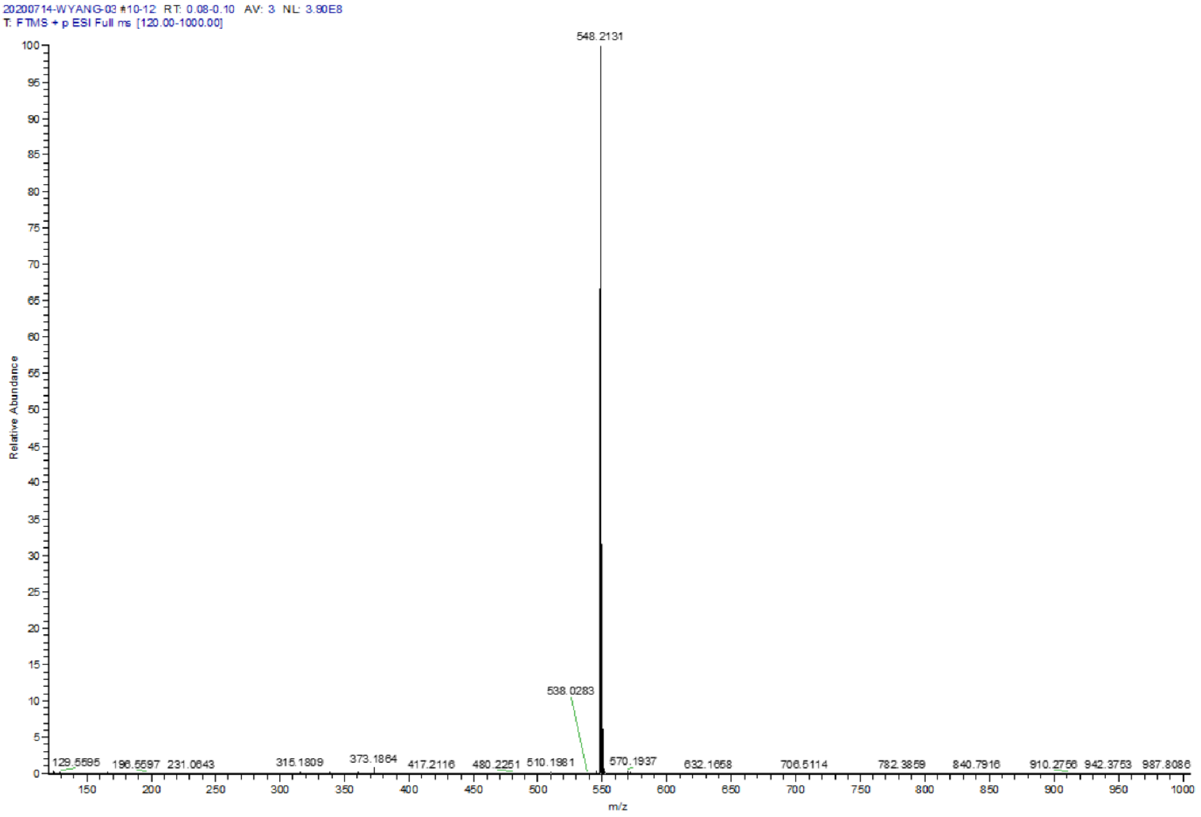

Figure S13. HRMS of compound 4.

Synthesis of R848-NTR-COOH: To synthesize R848-NTR-COOH, compound 4 was linked to carboxyl PEG-N_3 (Mw = 2000) *via* a copper (I)-catalyzed azide-alkyne cycloaddition (CuAAC) click reaction. In detail, compound 4 (30 mg) and carboxyl PEG-N_3 ((Mw = 2000, 100 mg) were dissolved in dry DMSO and t-butanol (v:v = 3:1) (1 mL), then sodium ascorbate (50 mg), TBTA (40 mg) and CuI (5 mg) were added to the solution under the protection of nitrogen, then sealed and stirred at room temperature for 24 h. Following completion, the mixture was dialyzed for 12 h against PBS buffer containing 10 mM EDTA-2Na, pH 7.4, followed by 24 h in deionized water and lyophilization to finally obtain R848-NTR-COOH (93.8 mg).

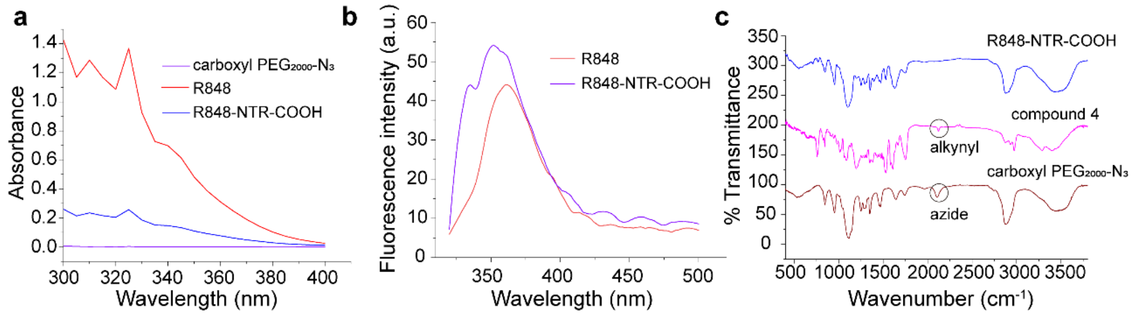

Figure S14. Characterizations of R848-NTR-COOH. a) UV-spectrum of R848-NTR-COOH, R848 and carboxyl PEG_{2000}-N_3. b) Fluorescence spectroscopy of R848-NTR-COOH and R848. c) FT-IR spectrums of R848-NTR-COOH, compound 4 and carboxyl PEG-N_3.

1.5 Synthesis of G5-CHC-R

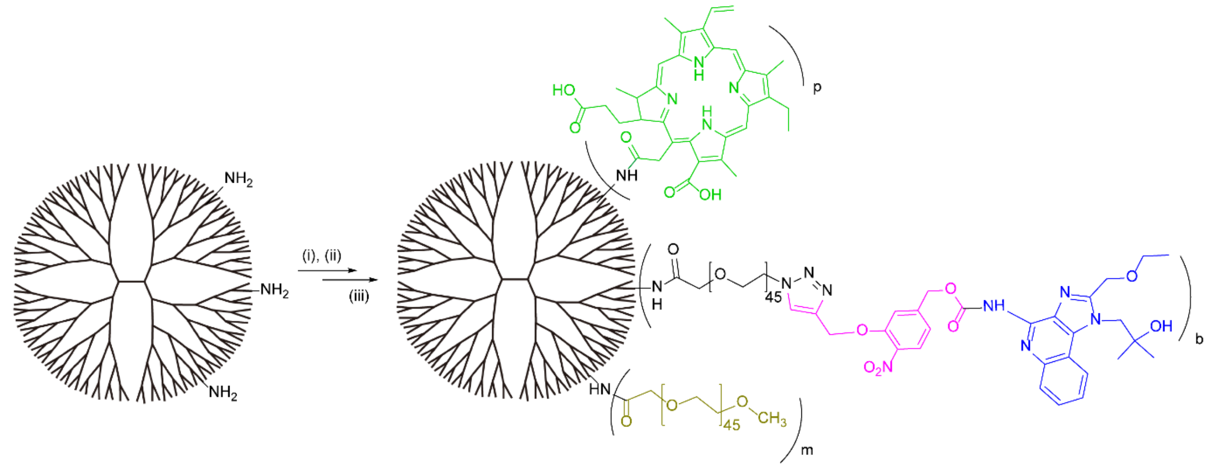

Figure S15. Structure and synthesis G5-CHC-R. Reagents and conditions: (i) CHC, HOBt, EDC; (ii) R848-NTR-COOH, NHS, EDC; (iii) Methoxyl PEG carboxyl (Mw = 2000), NHS, EDC. Conjugation numbers, p = 14-18 per G5, b = 17-20 per G5.

At room temperature, a mixture of CHC (5 mg), EDC (10 mg), and HOBt (8 mg) in anhydrous DMSO (1 mL) was stirred for 2 h. The above solution was then supplemented with

PAMAM G5 (10 mg) and DMAP (11 mg) which were dissolved in anhydrous DMSO in advance. The reaction was carried out at room temperature for 12 h. After that, a mixture of R848-NTR-COOH (20 mg), EDC (6 mg), and NHS (4 mg) was stirred at room temperature for 2 h in anhydrous DMSO (1 mL). This mixture was then added to the above solution along with 20 μL of DIPEA. Finally, a mixture of Methoxyl PEG carboxyl (Mw = 2000, 20 mg), EDC (6 mg), and NHS (4 mg) in anhydrous DMSO (1 mL) was stirred at room temperature for 2 h. This mixture was then added to the reaction solution previously described and supplemented with 20 μL of DIPEA. The reaction was carried out at room temperature for 12 h. To remove unreacted reagents and by-products, the reaction mixture was dialyzed (regenerated cellulose membrane, MWCO 10 KDa) against PBS. The obtained aqueous solution was the target product after filtration through a 0.22 μm polyvinylidene fluoride filter.

1.6 Synthesis of G5-CHC

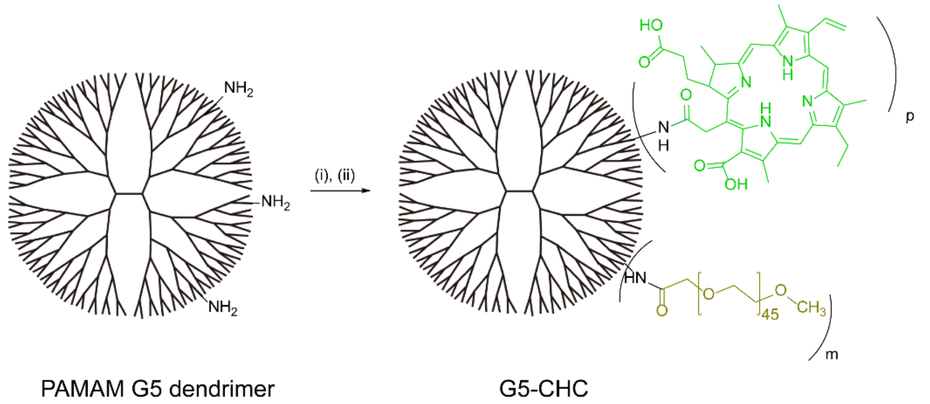

Figure S16. Structure and synthesis of G5-CHC. Reagents and conditions: (i) CHC, HOBt, EDC; (ii) Methoxyl PEG carboxyl (Mw = 2000), NHS, EDC. Conjugation numbers, p = 14-18 per G5.

A mixture of CHC (5 mg), EDC (10 mg), and HOBt (8 mg) in anhydrous DMSO (1 mL) was stirred at room temperature for 2 h. The above solution was then supplemented with PAMAM 5.0 (10 mg) and DMAP (11 mg) which were dissolved in anhydrous DMSO in advance. The reaction was stirred at room temperature for 12 h. Following that, a mixture of Methoxyl PEG carboxyl (Mw = 2000, 40 mg), EDC (11 mg), and NHS (7 mg) in anhydrous DMSO (1 mL) was stirred at room temperature for 2 h. This mixture was then added to the above solution along with 20 μL of DIPEA, and the reaction was stirred at room temperature for 12 h. Finally, the reaction mixture was dialyzed (regenerated cellulose membrane, MWCO 10 KDa) against PBS to remove unreacted reagents and by-products. The obtained aqueous solution was the target product after filtration through a 0.22 μm polyvinylidene fluoride filter.

1.7 Synthesis of G5-R

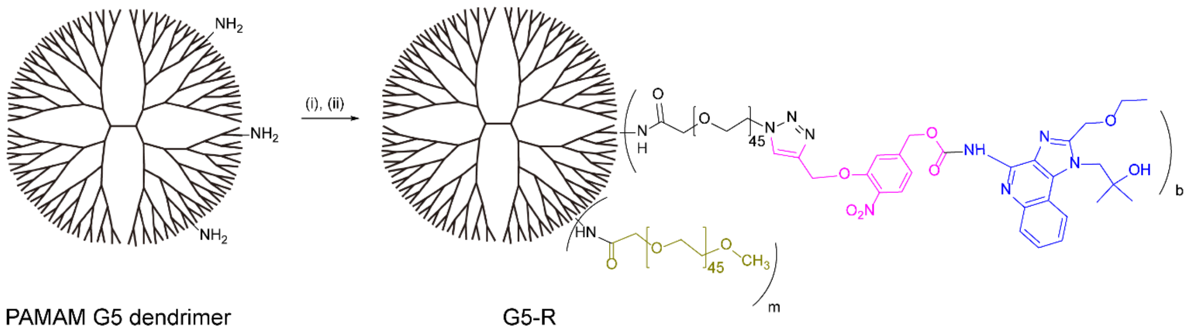

Figure S17. Structure and synthesis G5-R. (i) R848-NTR-COOH, NHS, EDC; (ii) Methoxyl PEG carboxyl (Mw = 2000), NHS, EDC. Conjugation numbers, b = 17-20 per G5.

At room temperature, a mixture of R848-NTR-COOH (20 mg), EDC (6 mg), and NHS (4 mg) in anhydrous DMSO (1 mL) was stirred for 2 h. The above solution was then supplemented with PAMAM 5.0 (10 mg) and DMAP (11 mg) which were dissolved in anhydrous DMSO in advance. The reaction was stirred at room temperature for 12 h. Subsequently, a mixture of Methoxyl PEG carboxyl (Mw = 2000, 25 mg), EDC (6 mg), and NHS (4 mg) in anhydrous DMSO (1 mL) was stirred at room temperature for 2 h. This mixture was then added to the above solution along with 20 μL of DIPEA, and the reaction was stirred at room temperature for 12 h. Finally, the reaction mixture was dialyzed (regenerated cellulose membrane, MWCO 10 KDa) against PBS to remove unreacted reagents and by-products. The obtained aqueous solution was the target product after filtration through a 0.22 μm polyvinylidene fluoride filter.

2. Supplementary Results

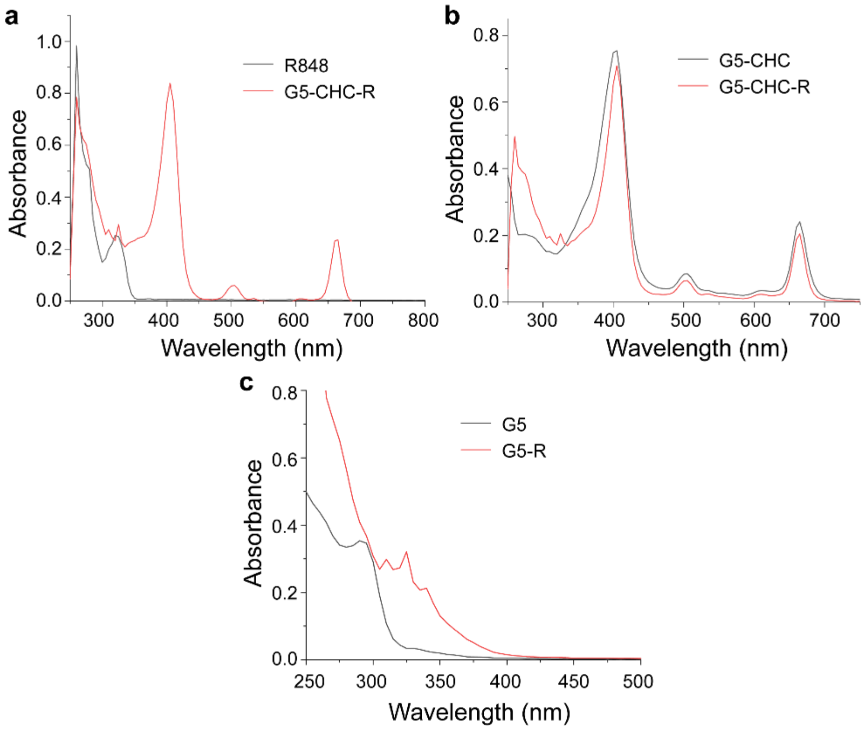

Figure S18. UV spectra of R848, G5-CHC-R, G5-CHC, G5-R and G5. a) UV-spectrum of G5-CHC-R and R848. b) UV-spectrum of G5-CHC-R and G5-CHC. c) UV-spectrum of G5 -R and G5.

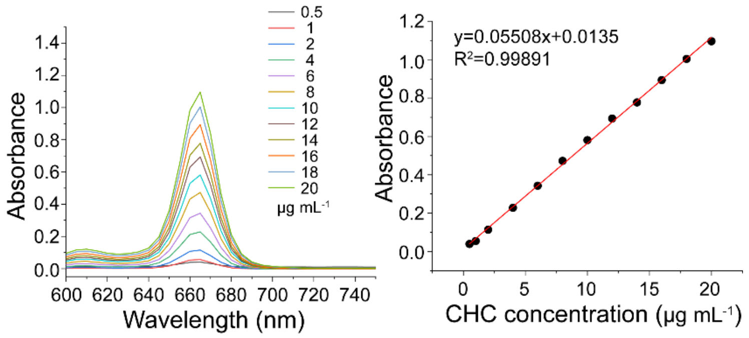

Figure S19. Determination of CHC concentration levels in G5-CHC and G5-CHC-R. UV-spectrum of CHC upon different concentrations (left). A standard curve based on absorbance at 665 nm. CHC dissolved in DMSO/DI water solutions (DMSO: DI water = 4:1) (right).

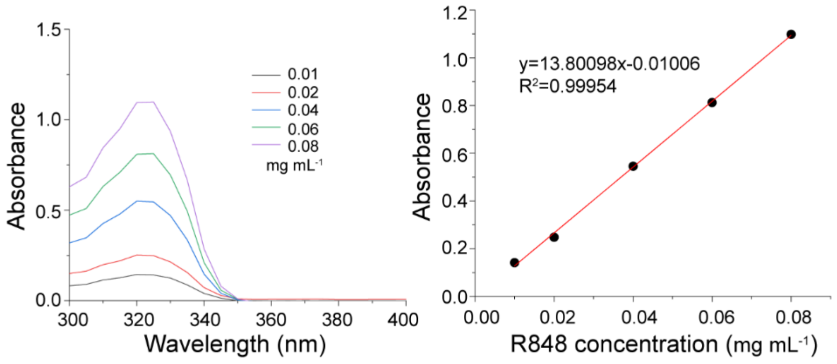

Figure S20. Determination of R848 concentration levels in G5-R and G5-CHC-R. UV-spectrum of R848 upon different concentrations (left). A standard curve based on absorbance at 325 nm. R848 dissolved in DMSO/DI water solutions (DMSO: DI water = 4:1) (right).

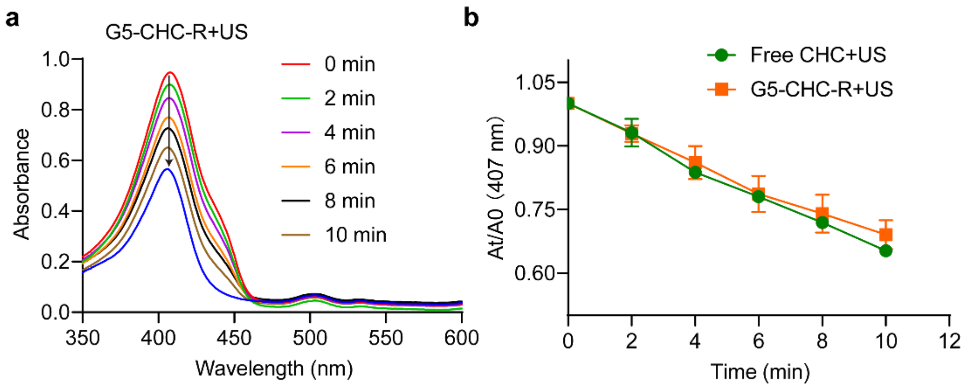

Figure S21. a) Absorption spectra of DPBF in the presence of G5-CHC-R with ultrasound irradiation for varied durations. b) Comparison of relative intensity of DPBF in free CHC and G5-CHC-R treated groups in the presence of US irradiation up to 10 min. Data are presented mean ± SD (n=3).

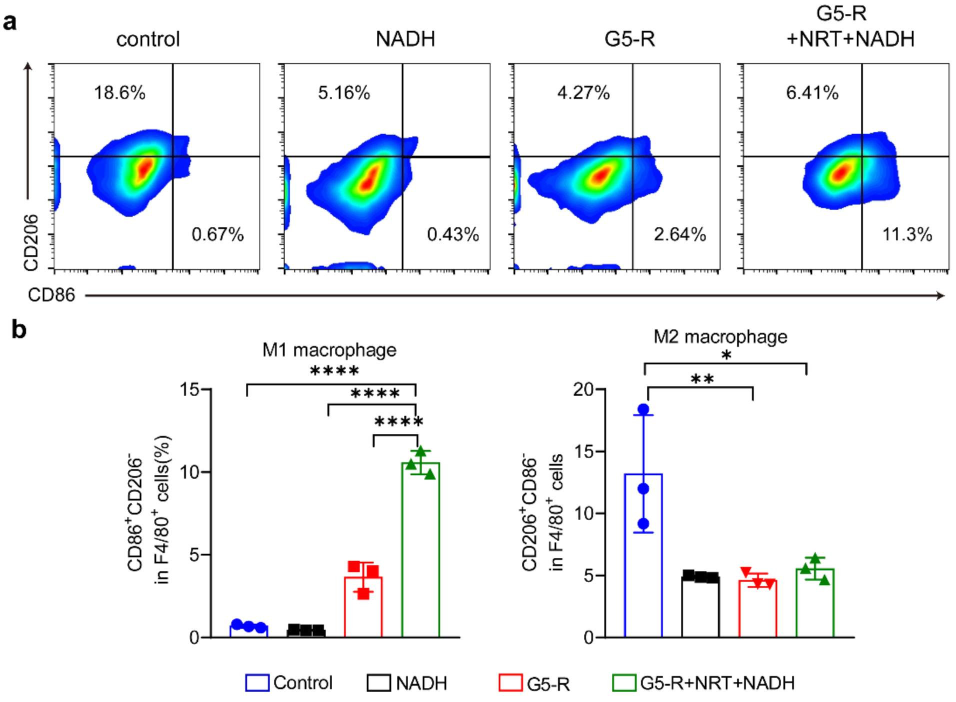

Figure S22. R848 release promote the phenotypic transformation of macrophages from M2 to M1. a) Flow cytometry analysis of *in vitro* macrophage polarization (gate on F4/80^{+} cells) at 24h after various treatments. b) Percentages of M1 (CD86^{+}CD206^{-}) and M2 (CD86^{-}CD206^{+}) macrophages in total macrophages. Data represented mean ± SD (n=3). Statistical significance was calculated *via* one-way ANOVA with Dunnett's multiple comparison test (b); *p*-value *$p < 0.05$, **$p < 0.01$, ***$p < 0.001$ and ****$p < 0.0001$.

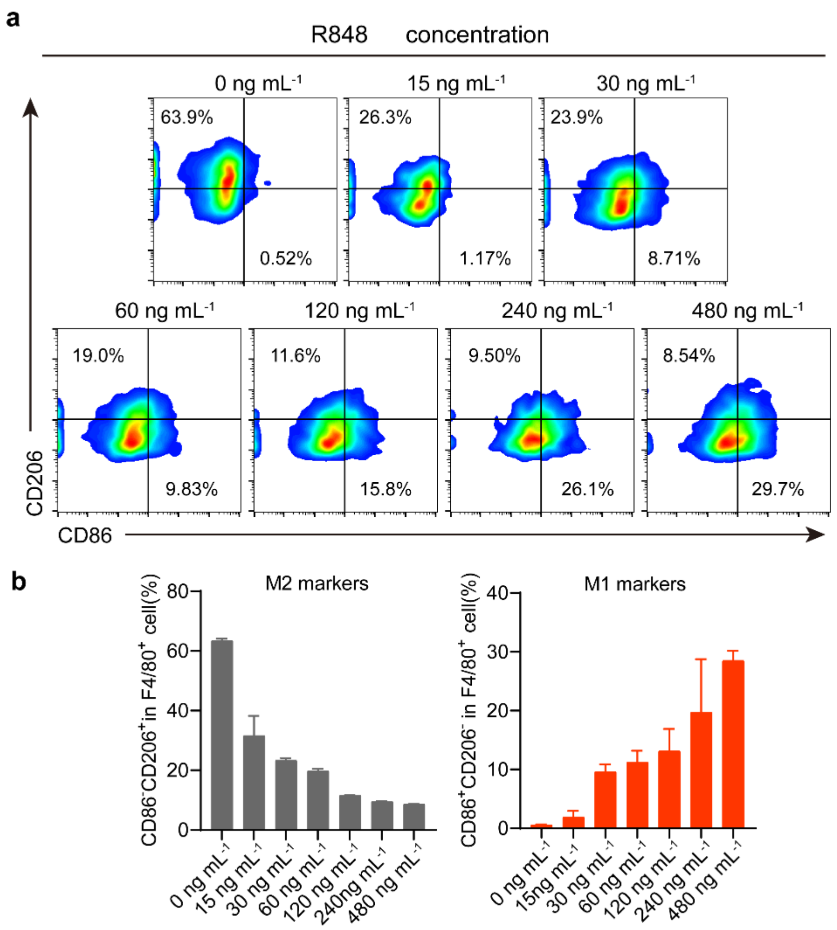

Figure S23. Repolarization of M2 macrophages to M1 phenotype by various concentrations of R848 (0, 15, 30, 60, 120, 240 and 480 ng/mL) in IL-4-conditioned BMDMs. Flow cytometry analysis (a) with quantification (b) of M1 ($CD86^+CD206^-$) and M2 ($CD86^-CD206^+$) macrophages in total macrophages. Data represented mean ± SD (n=3).

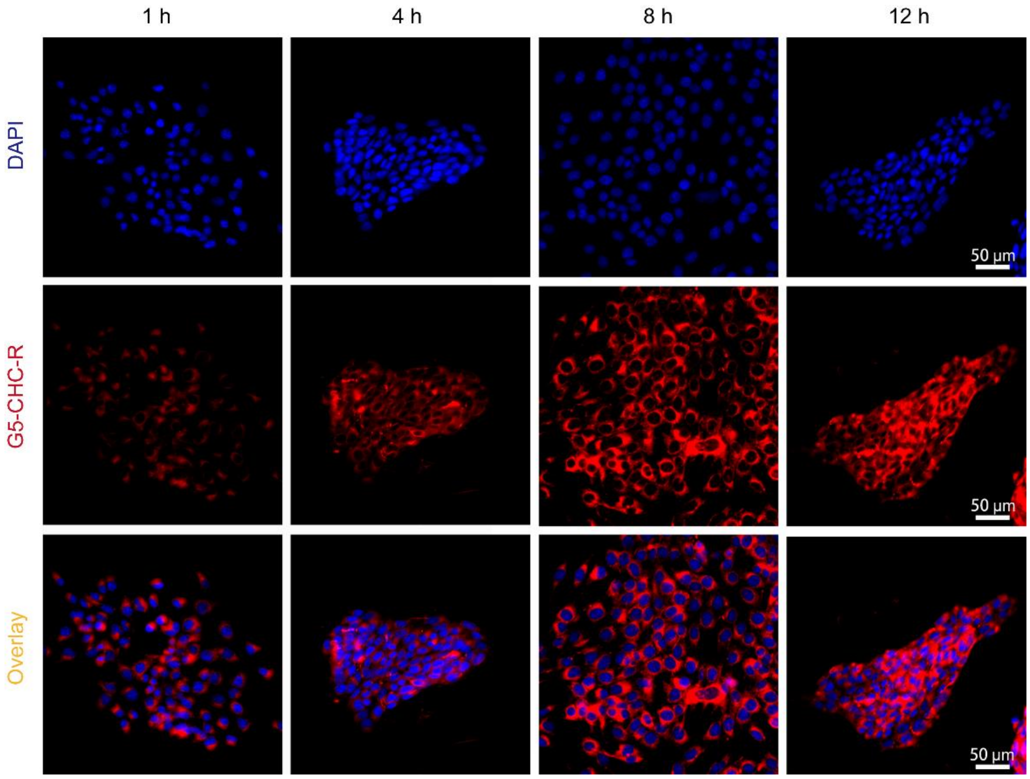

Figure S24. 4T1 uptake after treatment with G5-CHC-R at different time points. Fluorescence microscopic images of cellular uptake of G5-CHC-R by 4T1 cells after 1, 4, 8, and 12 h of incubation (Ex 590-650 nm, Em 662-738 nm, scale bar, 50 μm).

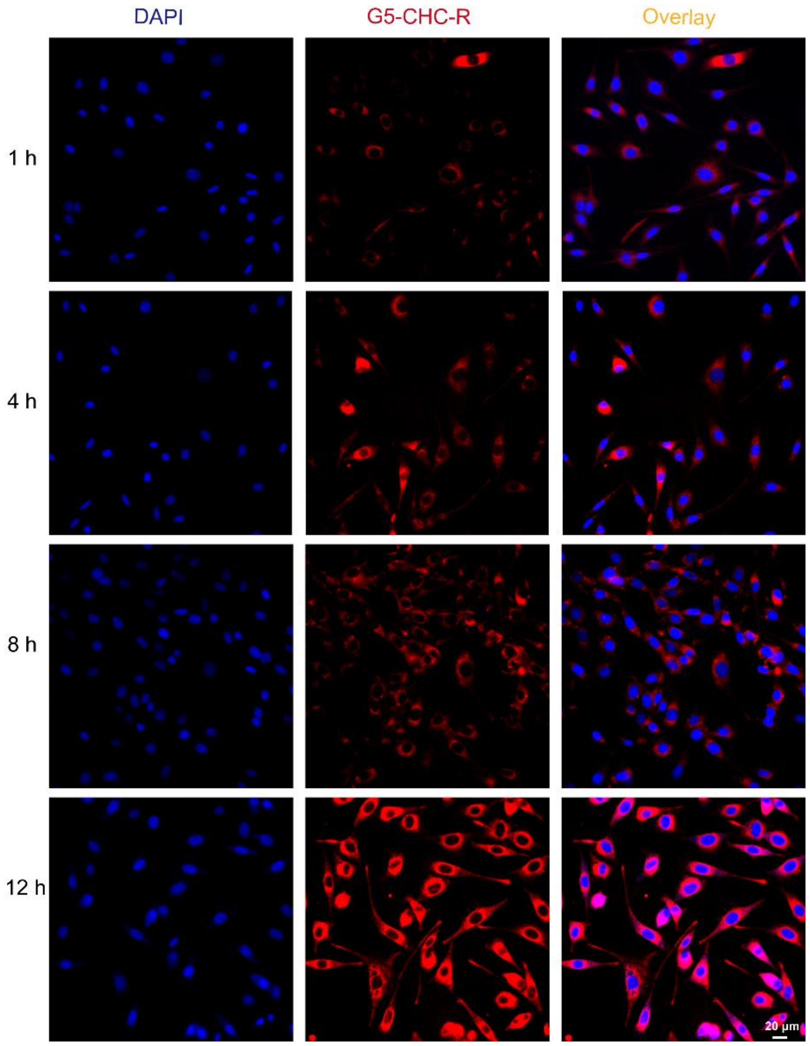

Figure S25. Pan02 uptake after treatment with G5-CHC-R at different time points. Fluorescence microscopic images of cellular uptake of G5-CHC-R by Pan02 cells after 1, 4, 8, and 12 h of incubation (Ex 590-650 nm, Em 662-738 nm, scale bar, 20 μm).

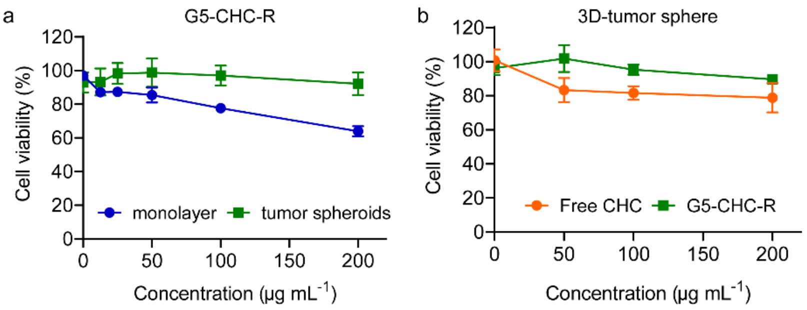

Figure S26. a) Cytotoxicities of G5-CHC-R against 4T1 cells in 2D culture and 3D tumor spheroids. b) Cytotoxicities of CHC and G5-CHC-R against 4T1 cells in 3D tumor spheroids. Data represented mean ± SD (n=4).

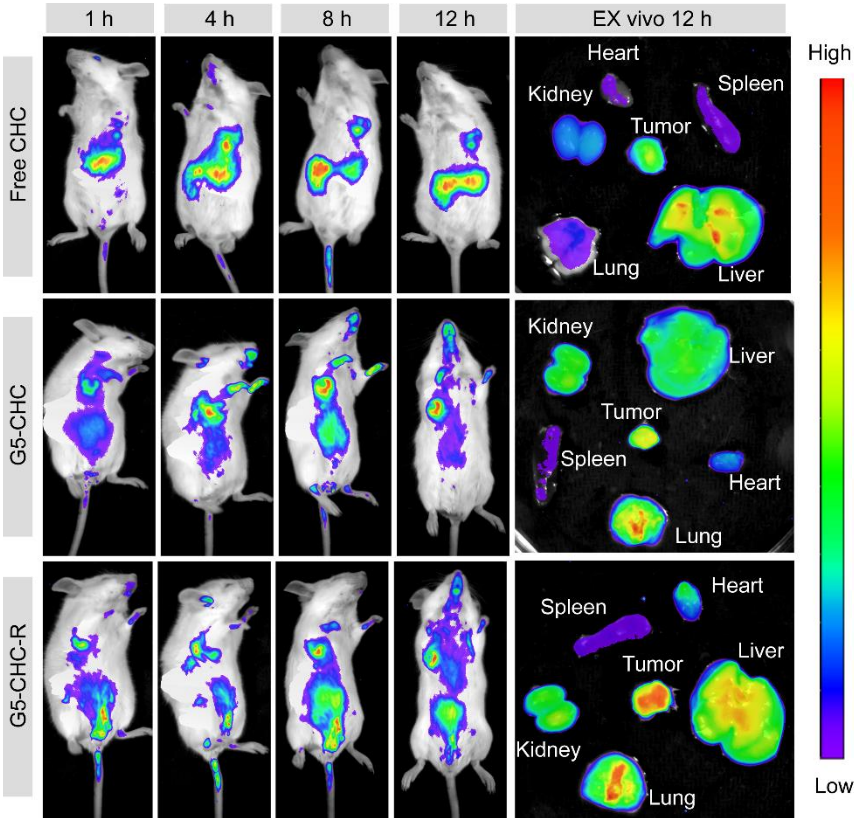

Figure S27. *In vivo* biodistribution of free CHC, G5-CHC and G5-CHC-R. Representative *in vivo* real-time fluorescence images of 4T1-bearing BALB/c mice after intravenous injection of free CHC, G5-CHC and G5-CHC-R at dosage of 15 mg CHC kg^{-1} bodyweight. Images were taken at 1, 4, 8, and 12 h post-injection. The *ex vivo* tissue images were taken at 12 h post-injection (Ex 665 nm, Em 700 nm).

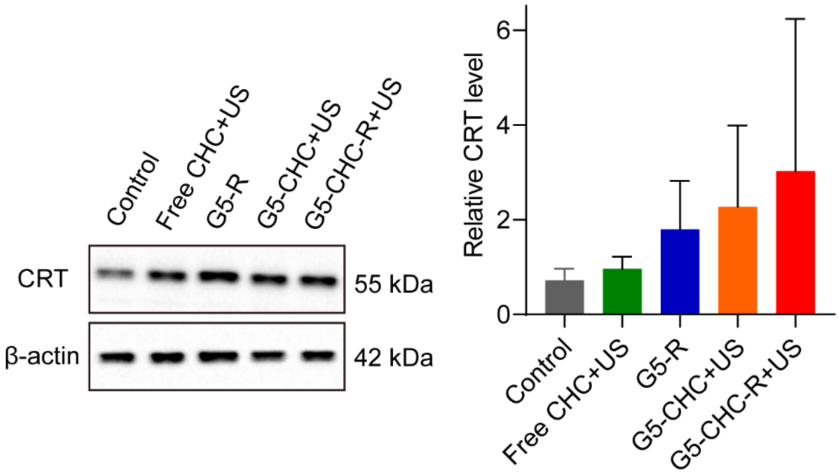

Figure S28. Representative CRT western blot image and quantify the expression of CRT by blot images. Data represented mean ± SD (n=2 or 3).

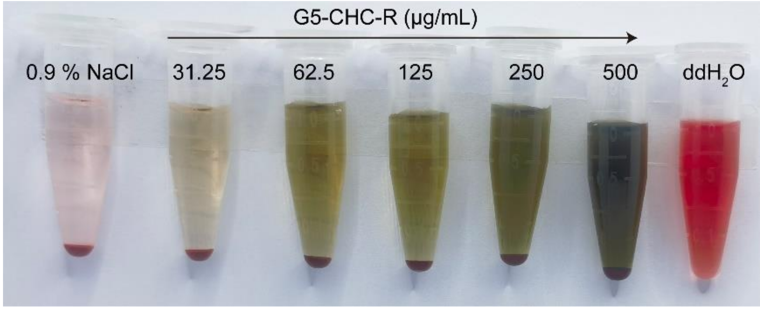

Figure S29. Hemolysis assay of G5-CHC-R at various concentrations.

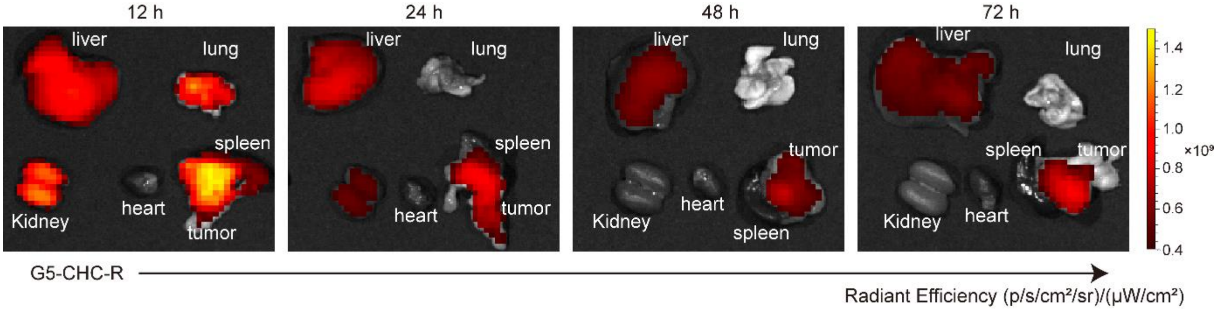

Figure S30. The *ex vivo* fluorescent images of major organs of mice at 12, 24, 48 and 72 h post-injected with G5-CHC-R.

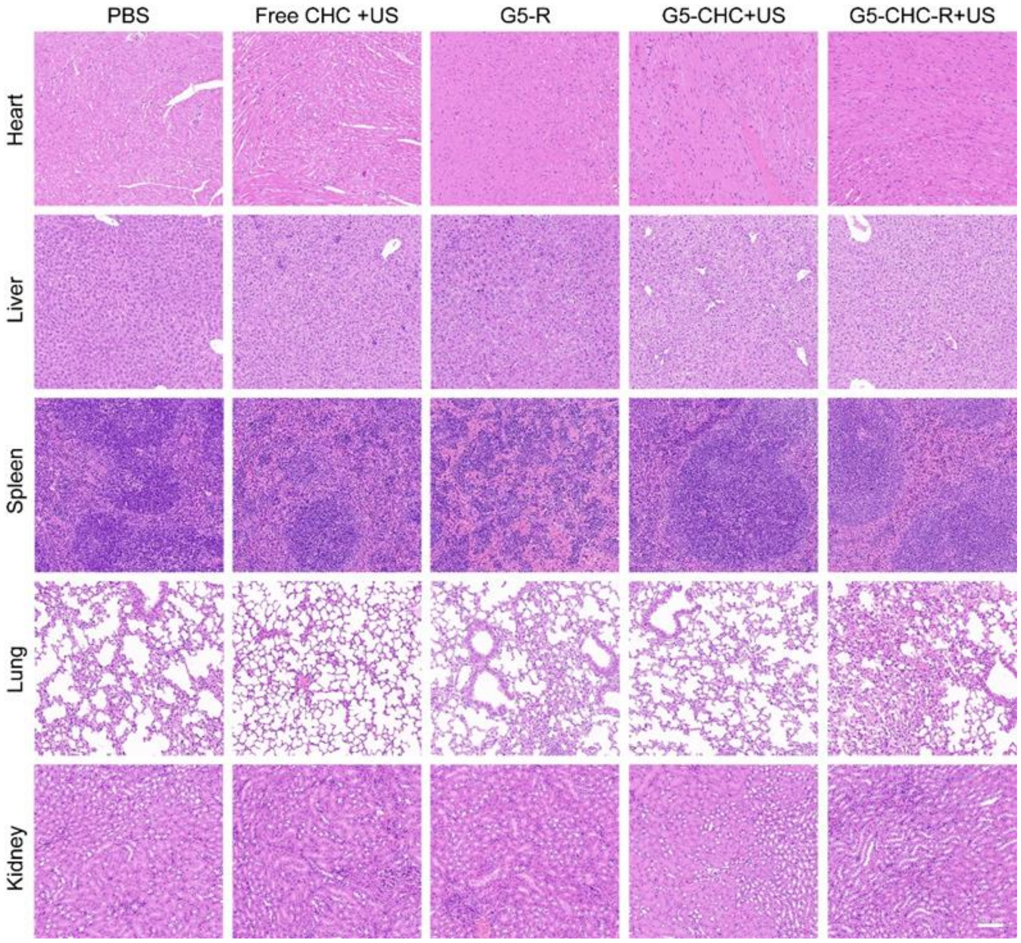

Figure S31. Histological analysis of different organs in Pan02 tumor-bearing mice after various treatments. No obvious signs of organ damage appeard in different groups treated mice. Scale bars, 100 μm.

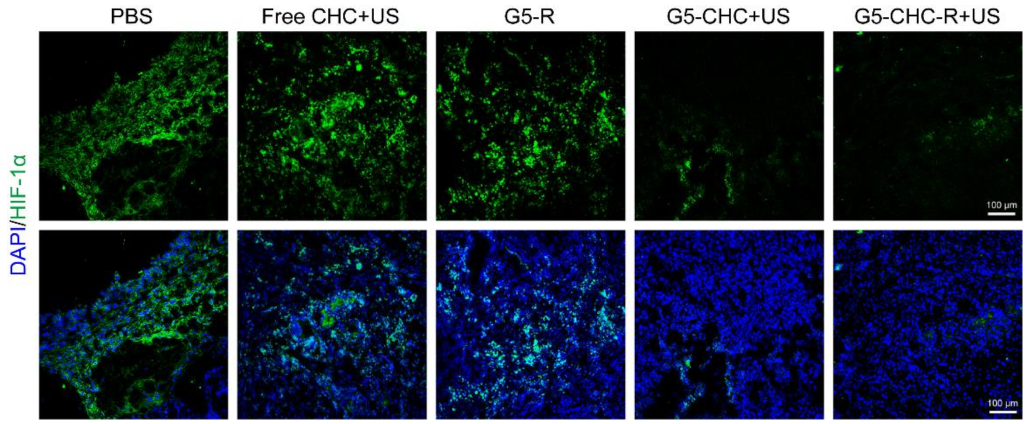

Figure S32. HIF-1α expression within tumor tissues after varied treatment. Immunofluorescence staining with HIF-1α in the excised tumor tissues after various treatments. Scale bar, 100 μm.

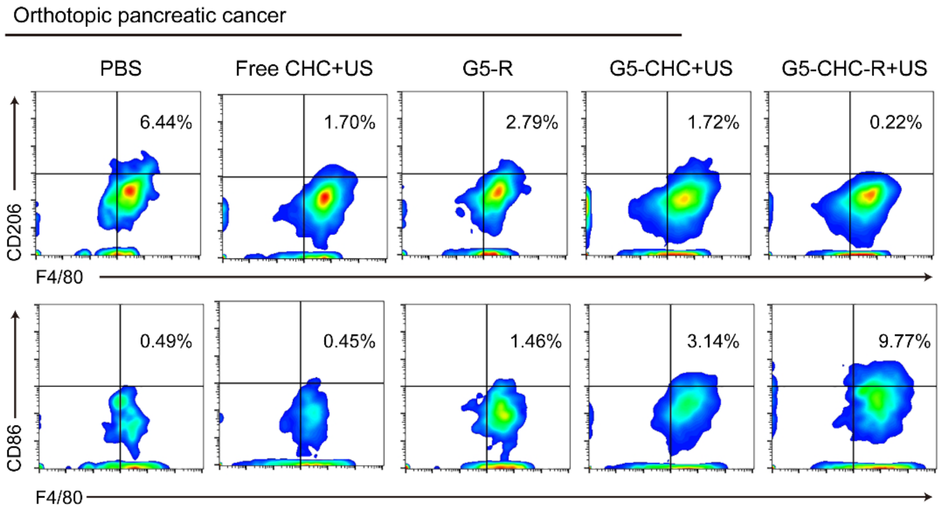

Figure S33. Representative dot plots of M2-phenotypic ($CD11b^+F4/80^+CD206^+$) and M1-phenotypic ($CD11b^+F4/80^+CD86^+$) macrophages in tumor tissues in the orthotopic pancreatic cancer model.

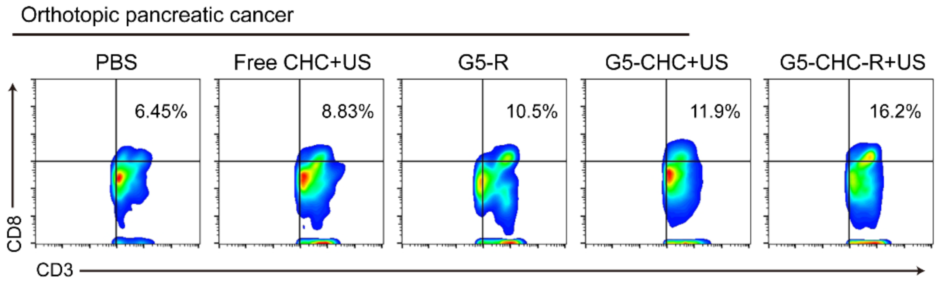

Figure S34. Representative dot plots of $CD8^+$T cells in tumor tissues in the orthotopic pancreatic cancer model.

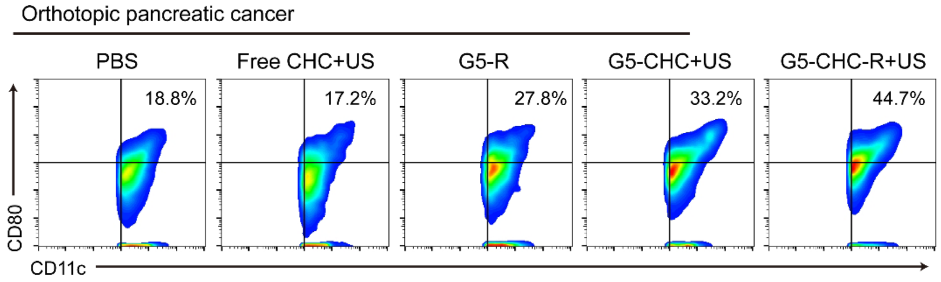

Figure S35. Representative dot plots of activated DCs ($CD80^+CD11c^+$) in tumor tissues in the orthotopic

pancreatic cancer model.

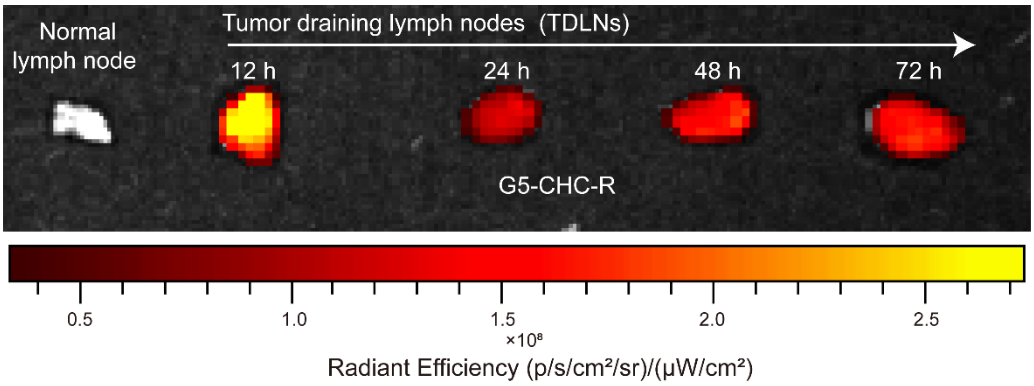

Figure S36. Representative fluorescent images of the excised lymph nodes (LNs) from normal mouse (left) and the tumor draining lymph nodes (TDLNs) from tumor-bearing mice (right) after i.v. administration of G5-CHC-R.

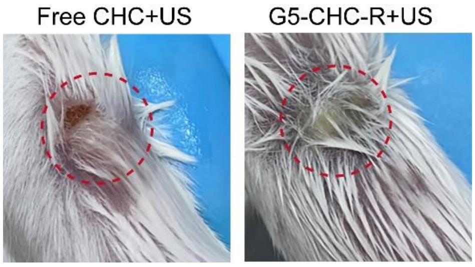

Figure S37. Tumor accumulation efficiency of the sono-nanovaccines. After three doses, the appearance of primary tumors of G5-CHC-R+US treated group were in dark green (left), but not Free CHC+US group (right).

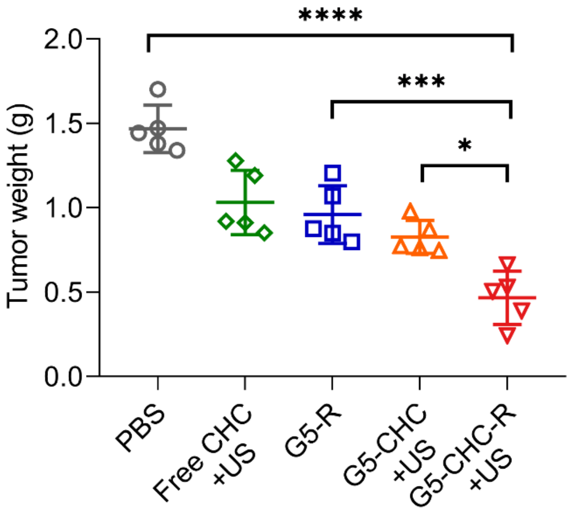

Figure S38. The average weights of excised 4T1 breast tumors. Data represented mean ± SD (n=5). Statistical significance was calculated *via* one-way ANOVA with Dunnett's multiple comparison test; *p*-value: $*p < 0.05$, $**p < 0.01$, $***p < 0.001$ and $****p < 0.0001$.

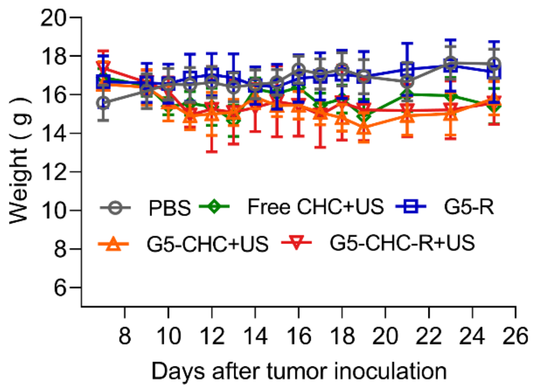

Figure S39. Dynamic body weights of 4T1-bearing mice in different groups during treatment. Data represented mean ± SD (n=5).

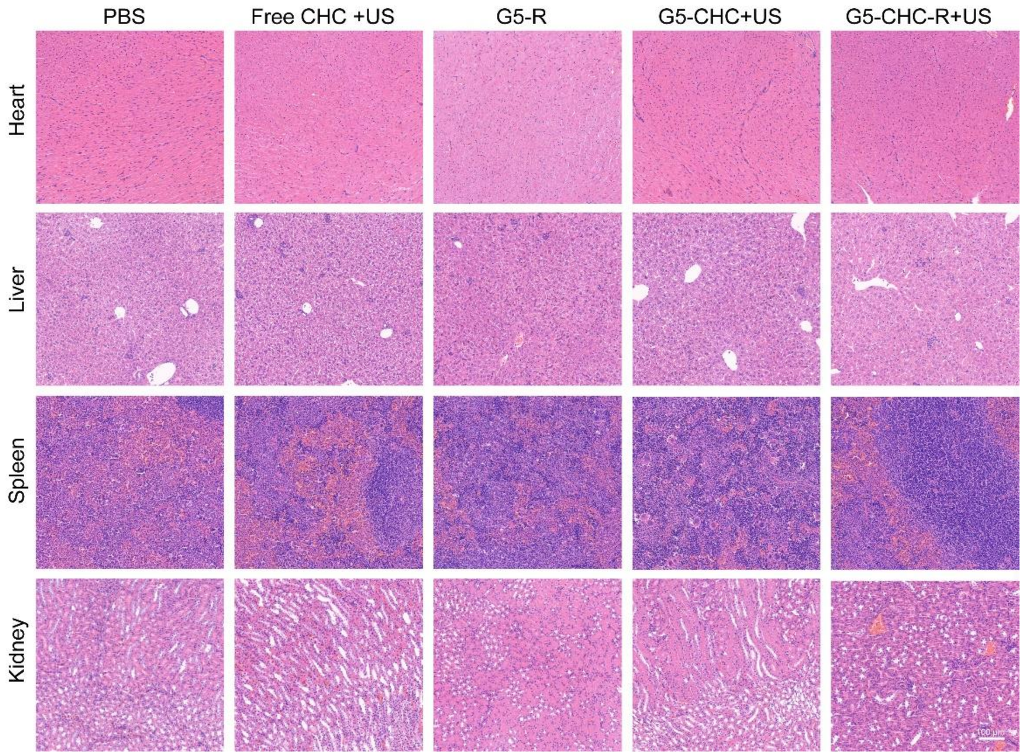

Figure S40. Histological analysis of different organs in 4T1 tumor-bearing mice after various treatments. No obvious signs of organ damage appeared in different groups treated mice. Scale bars, 100 μm.

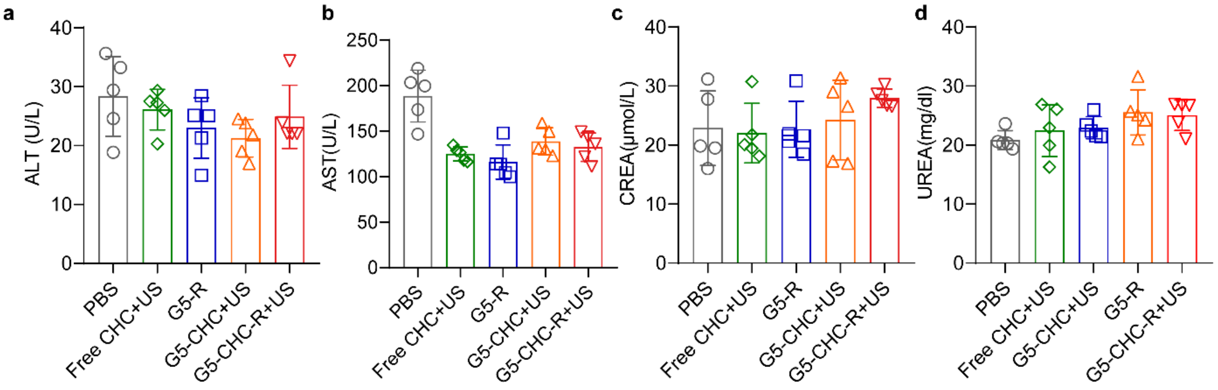

Figure S41. Important indicators of liver and kidney function after various treatments. a-d) Blood biochemical analysis of 4T1 tumor-bearing mice after administration of PBS, free CHC, G5-CHC, G5-R and G5-CHC-R. The levels of alanine aminotransferase (ALT) (a) and aspartate aminotransferase (AST) (b) indicated liver function, and creatinine (CREA) (c) and carbamide (UREA) (d) indicated renal function. Data represented mean ± SD (n=5).

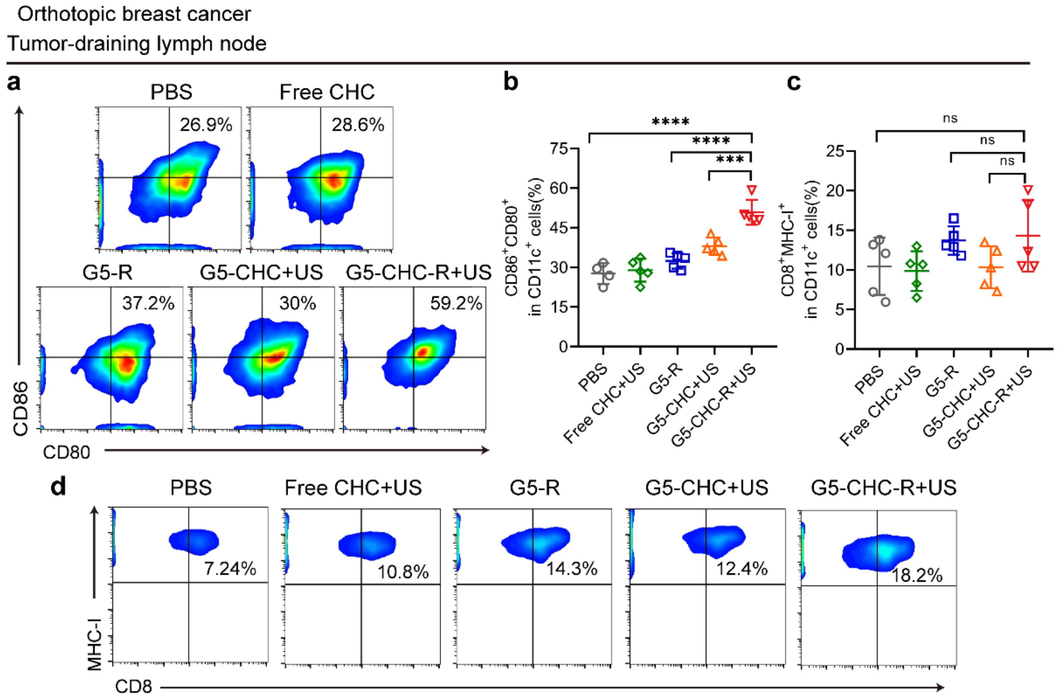

Figure S42. The free-field based whole-body ultrasound-driven nanovaccines enhances the antitumor immune response in TDLNs of 4T1 tumor-bearing mice. a-d) DCs in tumor draining lymph nodes were analysed for their activated status ($CD80^{+}CD86^{+}$) and cross-presenting capacities ($MHC\text{-}I^{+}CD8^{+}$), shown as representative dot plots (a, d) and percentage analysis (b, c) in 4T1 tumor-bearing mice after various treatments (n=5). Data represented mean ± SD. Statistical significance was calculated via Student's t test or one-way ANOVA with Dunnett's multiple comparison test (b, c); ns means no significant difference. p-value: $*p < 0.05$, $**p < 0.01$, $***p < 0.001$ and $****p < 0.0001$.

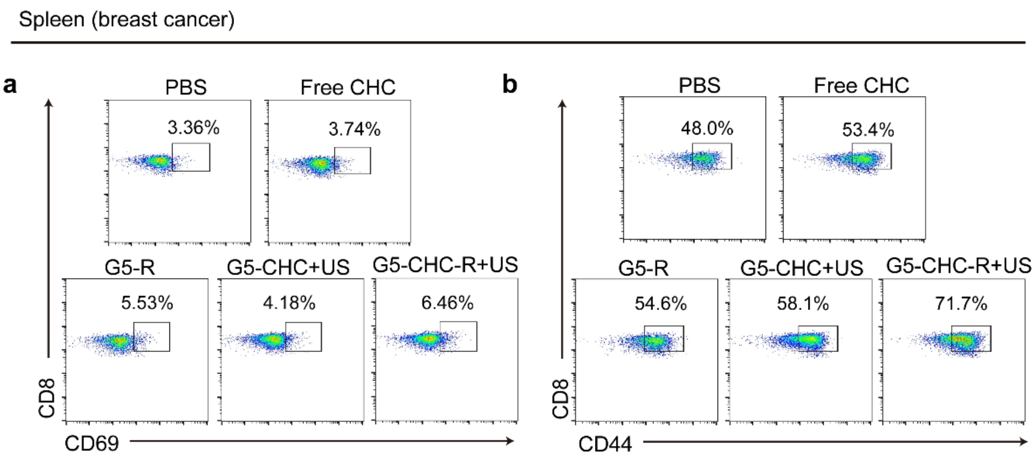

Figure S43. The free-field based whole-body ultrasound-driven nanovaccines enhances the frequency of memory $CD8^{+}$T and activated $CD8^{+}$T in spleen of 4T1 tumor-bearing mice. Representative dot plots of activated $CD8^{+}$ T cells ($CD69^{+}CD8^{+}$) (a) and memory-phenotypic ($CD44^{+}CD8^{+}$) (b) in spleens of 4T1 tumor-bearing mice after various treatments.

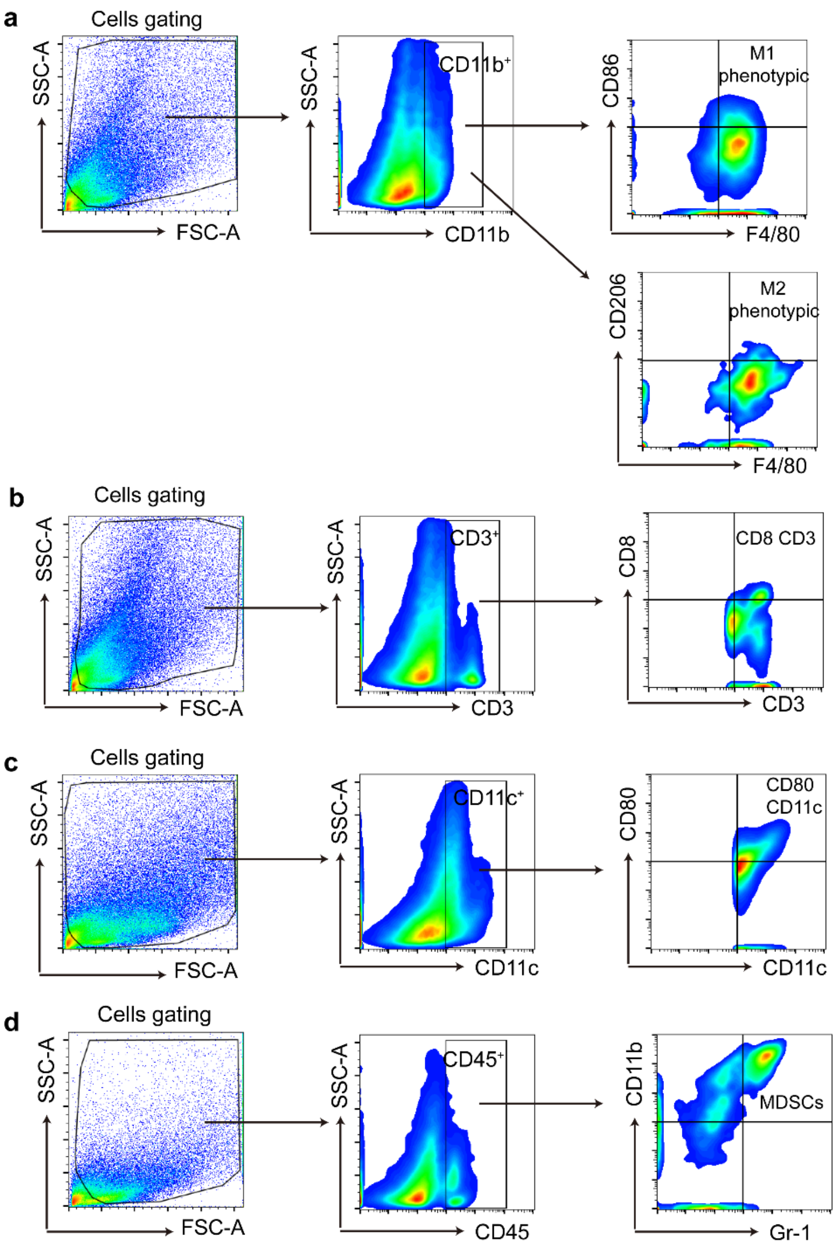

Figure S44. Gating strategies used for FACs analysis in the of pancreatic cancer mice. Gating strategy for identifying M1-phenotypic ($CD11b^{+}F4/80^{+}CD86^{+}$) and M2-phenotypic ($CD11b^{+}F4/80^{+}CD206^{+}$) macrophages (a), $CD8^{+}$ T cells (b), matured DCs ($CD11c^{+}CD80^{+}$) (c) and MDSCs ($CD11b^{+}Gr\text{-}1^{+}CD45^{+}$) (d).

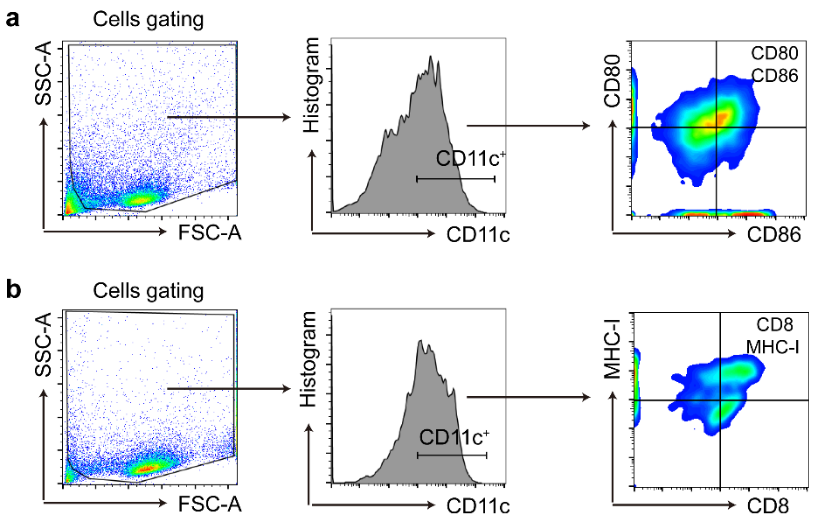

Figure S45. Gating strategies used for FACs analysis in the of TDLNs. Gating strategy for identifying matured DCs (CD11c^{+}CD80^{+}CD86^{+}) (a), cross-presenting DCs (CD11c^{+}CD8^{+}MHC-I^{+}) (b).

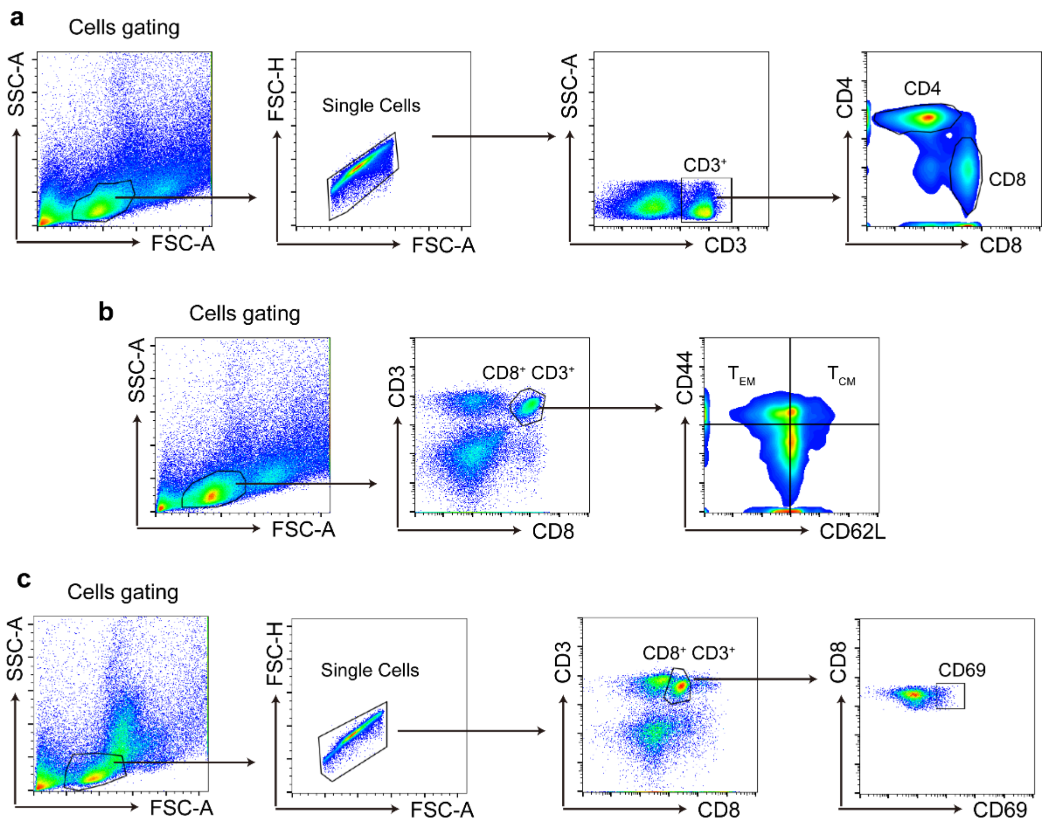

Figure S46. Gating strategies used for FACs analysis in the of splenocytes. Gating strategy for identifying CD8^{+} T cells (a), T_{EM} (CD3^{+}CD8^{+}CD44^{+}CD62^{-}) and T_{CM} (CD3^{+}CD8^{+} CD44^{+}CD62^{+}) (b), activated CD8^{+} T cells (CD3^{+}CD8^{+} CD69^{+}).

Table S1. Mouse antibodies for flow cytometry and FACS.

Antigen	Clone	Fluorophore	Source	Dilution ration
CD11c	N418	PE/Cy7	BioLegend	1:100
CD86	GL-1	APC, PerCP/Cy5.5	BioLegend	1:100
CD80	B7-1	APC, PE	BioLegend	1:100
CD3	17A2	PE, FITC	BioLegend	1:100
CD4	RM4-5	APC	BioLegend	1:100
CD8	53-6.7	FITC, PerCP, APC	BioLegend	1:100
CD69	H1.2F3	FITC, PE	BioLegend	1:100
CD62L	MEL-14	FITC, PerCP/Cy5.5	BioLegend	1:100
CD44	IM7	FITC	BioLegend	1:100
MHC-I	34-1-2S	APC	BioLegend	1:100
CD11b	M1/70	PE, FITC	BioLegend	1:100
F4/80	BM8	FITC, PE	BioLegend	1:100
CD206	C068C2	PE/Cy7, APC	BioLegend	1:100
Gr-1	RB6-8C5	APC	BioLegend	1:100
CD49b	HMa2	Alexa.Fluor®488, PE	BioLegend	1:100
CD45	30-F11	FITC	BioLegend	1:100

Table S2. Tissue immunofluorescence antibodies.

Antigen	Catalog number	Species	Source
Calreticulin (1:400)	#12238	Rabbit lgG	Cell Signaling
HMGB1(1:100)	#3935	Rabbit	Cell Signaling
CD8(1:500)	66868-1-lg	Mouse	Proteintech
iNOS(1:100)	18985-1-AP	Rabbit	Proteintech
CD206(1:200)	60143-1-lg	Mouse	Proteintech
IFN-γ(1:200)	507801	Mouse	BioLegend
Calreticulin (1:400)	#12238	Rabbit lgG	Cell Signaling

Table S3. Tissue immunofluorescence antibodies.

Antigen	Catalog number	Source	Dilution ration
Fluorescein(FITC) conjugated Affinipure Goat Anti-Rabbit lgG	SA00001-2	Proteintech	1:50
Goat Anti-Rabbit lgG(H+L), Coralite594 conjugate	SA00013-4	Proteintech	1:100
Goat Anti-Mouse lgG(H+L), Coralite488 conjugate	SA00013-1	Proteintech	1:100
